# *Escherichia coli*‐Induced cGLIS3‐Mediated Stress Granules Activate the NF‐κB Pathway to Promote Intrahepatic Cholangiocarcinoma Progression

**DOI:** 10.1002/advs.202306174

**Published:** 2024-02-17

**Authors:** Feng‐Ping Kang, Zhi‐Wen Chen, Cheng‐Yu Liao, Yong‐Ding Wu, Ge Li, Cheng‐Ke Xie, Hong‐Yi Lin, Long Huang, Yi‐Feng Tian, Zu‐Wei Wang, Shi Chen

**Affiliations:** ^1^ Shengli Clinical Medical College of Fujian Medical University Fuzhou 350001 China; ^2^ Department of Hepatobiliary Pancreatic Surgery Fujian Provincial Hospital Fuzhou 350001 China; ^3^ Department of Hepatobiliary Surgery and Fujian Institute of Hepatobiliary Surgery Fujian Medical University Union Hospital Fuzhou 350001 China; ^4^ Fujian Key Laboratory of Geriatrics Fujian Provincial Hospital Fuzhou 350001 China

**Keywords:** circGLIS3, *Escherichia coli*, Icaritin, intrahepatic cholangiocarcinoma, NF‐κB, stress granules

## Abstract

Patients with concurrent intrahepatic cholangiocarcinoma (ICC) and hepatolithiasis generally have poor prognoses. Hepatolithiasis is once considered the primary cause of ICC, although recent insights indicate that bacteria in the occurrence of hepatolithiasis can promote the progression of ICC. By constructing in vitro and in vivo ICC models and patient‐derived organoids (PDOs), it is shown that *Escherichia coli* induces the production of a novel RNA, circGLIS3 (cGLIS3), which promotes tumor growth. cGLIS3 binds to hnRNPA1 and G3BP1, resulting in the assembly of stress granules (SGs) and suppression of hnRNPA1 and G3BP1 ubiquitination. Consequently, the IKKα mRNA is blocked in SGs, decreasing the production of IKKα and activating the NF‐κB pathway, which finally results in chemoresistance and produces metastatic phenotypes of ICC. This study shows that a combination of Icaritin (ICA) and gemcitabine plus cisplatin (GP) chemotherapy can be a promising treatment strategy for ICC.

## Graphical abstract

Patients with intrahepatic cholangiocarcinoma (ICC) complicated with hepatolithiasis have a poor prognosis. This study reveals that bacteria play a role in this process. *Escherichia coli* induces cGLIS3 expression and promotes tumor progression in ICC. cGLIS3 triggers the assembly of stress granules, activates the NF‐κB pathway, and causes chemoresistance. Icaritin (ICA) combined with Gemcitabine/Cisplatin (GP) is a promising therapy for ICC.

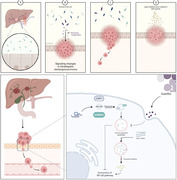

## 1 Introduction

Intrahepatic cholangiocarcinoma (ICC) involving the secondary branches of the bile duct is associated with a poor prognosis.^[^ ^1^ ^]^ It is the second most common malignant tumor of the biliary tract and accounts for 10–15% of primary liver cancers. In recent years, the incidence of ICC has significantly increased worldwide.^[^ ^1^, ^2^ ^]^ However, the mechanisms underlying their occurrence and development are not completely understood. Previous research has shown that hepatolithiasis, a condition in which stones are formed in the liver, is a high‐risk factor for ICC, particularly in China.^[^ ^3^ ^]^ Patients with concurrent ICC and hepatolithiasis often have poor prognosis.^[^ ^4^ ^]^ While hepatolithiasis was once considered the primary cause of ICC, recent studies suggest that bacteria play a significant role in the formation of hepatolithiasis and the progression of ICC.^[^ ^5^ ^]^ Bacteria facilitate the occurrence of hepatolithiasis by interacting with host bile tissue,^[^ ^6^ ^]^ which leads to hepatolithiasis‐induced cholestasis, bacterial proliferation, and inflammatory responses. These events promote ICC progression. Clinical data indicate that patients with ICC and hepatolithiasis often exhibit an imbalance in the biliary microbiota, including an overgrowth of *Escherichia coli* (*E. coli*), which may promote the malignant behavior of ICC cells. While previous research has confirmed the role of *E. coli* in carcinogenesis,^[^ ^7^, ^8^, ^9^ ^]^ very few studies have investigated the oncogenic behavior of *E. coli* in ICC.^[^ ^10^ ^]^

Stress granules (SGs) play a crucial role in cell survival during bacterial infection by responding to microenvironmental stimuli.^[^ ^11^, ^12^, ^13^ ^]^ These granules protect cells from stress‐induced damage and death by sequestering numerous ribonucleoprotein particles (RNPs) and signaling proteins.^[^ ^14^, ^15^ ^]^ This mechanism is essential for promoting cell survival under physiological and pathological conditions.^[^ ^16^ ^]^ However, it has been reported that cancer cells exploit SGs to facilitate distant metastasis and tumor chemoresistance.^[^ ^12^ ^]^

Circular RNAs (circRNAs) can regulate cellular stress. Some circRNAs play crucial roles in maintaining homeostasis, whereas others when dysregulated, may enhance the stress response to adapt to chronic stress during cancer development.^[^ ^17^ ^]^ Several studies have reported on the promotion and degradation of SGs by circRNAs. For instance, circTBC1D14 can initiate a cascade reaction of SG‐associated proteins and enhance lysosome‐related autophagy to recognize and regulate SG elimination.^[^ ^18^ ^]^ Similarly, circVAMP3 promotes SGs formation by driving the phase separation of CAPRIN1. In addition, circVAMP3 negatively regulates the proliferation and metastasis of HCC cells.^[^ ^19^ ^]^ Note that eIF4A3 is an important splicing factor in the circularization of circRNAs^[^ ^20^ ^]^ and has been reported to be associated with SGs formation.^[^ ^21^ ^]^ However, the specific mechanisms underlying the regulation of SGs remain unclear. Therefore, it is necessary to investigate the role of eIF4A3 in the regulation of SGs.

The NF‐κB pathway is crucial for regulating various gastrointestinal malignancies,^[^ ^22^ ^]^ including ICC.^[^ ^23^ ^]^ It is involved in the regulation of gene expression, epithelial‐mesenchymal transition, and chemoresistance. CircRNAs regulate the NF‐κB pathway by promoting the activation or inhibition of downstream pathways.^[^ ^24^ ^]^ However, it has not yet been clearly established whether circRNA‐mediated regulation of the NF‐κB pathway plays a role in SGs formation.

This study investigated the interplay between *E. coli* and the malignant progression of ICC and aim to investigate the mechanisms by which SGs assemble in ICC cells in a microenvironment containing *E. coli*. We showed that *E. coli* promotes the production of a novel RNA, circGLIS3 (cGLIS3), which promotes SG assembly by regulating the interaction and ubiquitination of hnRNPA1 and G3BP1. As a result, IKKα mRNA is blocked by SGs and then NF‐κB pathway is activated, leading to tumor chemoresistance and invasive migratory phenotypes. Our results suggest that the combination of Icaritin (ICA) and GP chemotherapy is a promising treatment strategy for ICC.

## 2 Results

### 2.1 Stimulation by *E. coli* Promotes the Progression of ICC and the Assembly of SGs

**Figure 1:**
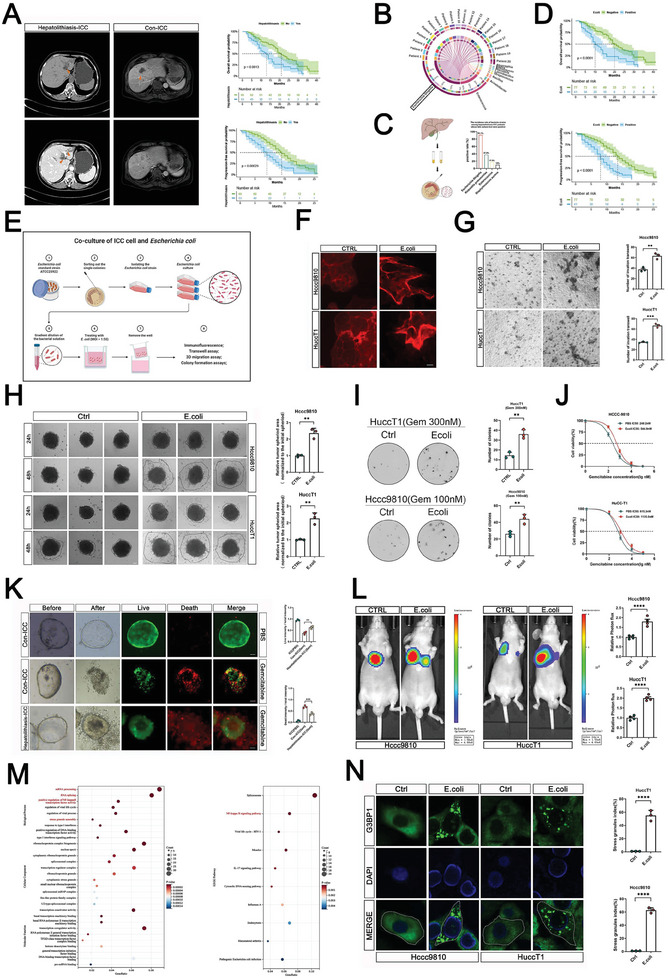
Stimulation of *E. coli* promotes the malignant progression of ICC and the assembly of SGs. A) Right:CT images of hepatolithiasis‐ICC patients and Con‐ICC patients. Left: The Kaplan–Meier survival analysis (overall survival probability and progression‐free survival probability) between hepatolithiasis‐ICC patients and Con‐ICC patients. B) 16S rRNA sequencing analysis of the proportion of bacterial microbiota in the bile of hepatolithiasis‐related ICC patients (*n* = 20). C) The diagram of bile culture test and the incidence rates of different bacteria in ICC patients whose bile bacterial culture test were positive (*n* = 45). D) The Kaplan–Meier survival analysis (overall survival probability and progression‐free survival probability) between ICC patients with *E. coli* positive according to bile culture test and ICC patients with *E. coli* negative. E) Diagram of indirect co‐culture model of *E. coli* and ICC cells. F) Confocal microscopy of cytoskeleton alterations based on immunofluorescence staining of F‐actin via phalloidin (red) in Hccc9810 and HuccT1 cells. Scale bar: 25 µm. G) Transwell assay of HuccT1 and Hccc9810 cells that treated by PBS and indirectly co‐cultured with *E. coli*; quantified in the right panel (*n* = 3). H) 3D migration assay of HuccT1 and Hccc9810 cells that treated by PBS and indirectly co‐cultured with *E. coli*; quantified in the right panel (*n* = 3). I) Colony formation assays of Hccc9810 and HuccT1 cells that treated by PBS and indirectly co‐cultured with *E. coli* further treated with gemcitabine. Representative colony formation images are shown. The numbers of colonies are summarized on the right (*n* = 3). J) Effect of treatment with *E. coli* or PBS, as observed through IC50 experiment. K) Representative images of the Con‐ICC and hepatolithiasis‐ICC PDOs after gemcitabine treatments. Live/Dead cell viability assay showing live cells stained with Calcein‐AM (green) and dead cells with EthD‐1 (red) (*n* = 3). L) Hccc9810 cells were indirectly co‐cultured with *E. coli* or PBS. After that Hccc9810 cells were injected into nude mice via the tail vein. Representative images and statistical analysis of in vivo lung bioluminescence in the lung metastasis model with indicated treatments (*n* = 4). M) GO (left) and KEGG (right) enrichment analyses of differentially expressed genes identified by RNA‐seq in HuccT1 cells co‐cultured with PBS or *E. coli*. N) Immunofluorescence of SGs (G3BP1) in Hccc9810 and HuccT1 cells treated with *E. coli*. Quantification of the percentage of cells with SGs (G3BP1) in the right panel. *n* = 3, Scale bar, 10 µm. (G–I,K–L,N) For bar and line graphs, data represent mean ± SEM. (G–I,L,N) Unpaired two‐tailed Student's *t*‐tests. J) Two‐way analysis of variance (ANOVA) with Bonferroni's post‐hoc test. K) One‐way ANOVA with Tukey's post‐hoc test. NS indicates no significance; ^**^ *p* < 0.01, ^***^ *p* < 0.001, and ^****^ *p* < 0.0001.

It has recently been reported that patients with concurrent ICC and hepatolithiasis have worse prognosis, which is consistent with our patient data (**Figure** **1**A; Table S1, Supporting Information). Hepatolithiasis typically results from a bacterial infection.^[^ ^24^ ^]^ We hypothesized that the microbiome of common hepatolithiasis, which usually exists in bile, may play a role in the development of ICC. Thus, we conducted a 16S rRNA sequencing analysis of bile from patients with hepatolithiasis‐related ICC and found that *E. coli* had the highest proportion among all identified bacteria (Figure 1B). Furthermore, we found that *E. coli* possessed the highest incidence rate in ICC patients whose bile bacterial culture tests were positive, according to our clinical data (Figure 1C). Notably, *E. coli*‐positive ICC patients have poorer overall survival (OS) and progression‐free survival (PFS) (Figure 1D; Tables S2,S3, Supporting Information). Furthermore, these hepatolithiasis‐related ICC patients exhibited significant changes in Interleukin‐6 (IL‐6) levels in the bile, which is consistent with studies showing a substantial role of IL‐6 in ICC progression^[^ ^25^, ^26^ ^]^ (Figure S1A, Supporting Information). We also found increased IL‐6 levels in the tumor homogenate from the subcutaneous model injected with Huccc9810 cells pretreated with *E. coli* and in the co‐culture medium derived from ICC cells indirectly co‐cultured with *E. coli* (Figure S1B, Supporting Information). Subsequently, we established an in vitro indirect co‐culture model of *E. coli* and ICC to observe the impact of this environment on tumor progression (Figure 1E). The ICC cells in the co‐culture group exhibited a more invasive phenotype based on the F‐actin staining assay (Figure 1F), enhanced migratory ability through transwell and 3D migration assays (Figure 1G,H), and increased resistance to gemcitabine (Figure 1I,J). Using the constructed patient‐derived organoid (PDO) model, we found that PDO derived from patients with both ICC and hepatolithiasis exhibited stronger resistance to gemcitabine (Figure 1K). In vivo experiments also confirmed that *E. coli*‐stimulated ICC cells had greater metastatic capacity (Figure 1L). To elucidate the underlying molecular mechanisms, we conducted an RNA‐seq analysis of ICC cells treated with PBS and indirectly co‐cultured with *E. coli* to identify enriched gene ontology (GO) and Kyoto Encyclopedia of Genes and Genomes (KEGG) terms. Classical inflammatory pathways such as NF‐κB were found to be enriched among the differentially expressed genes. Interestingly, we found significant enrichment in RNA processing and an increase in SGs assembly in GO terms, suggesting a potential association between RNA processing and SGs assembly that leads to the progression of ICC cells (Figure 1M). In vitro investigation also revealed that ICC indirectly co‐cultured with *E. coli* formed more SGs than the control group (Figure 1N). Notably, we found that IL6 plays an important role in promoting ICC progression caused by stimulation with *E. coli* (Figure S1C–G, Supporting Information).

### 2.2 *E. coli* Promotes SGs Formation Through the Upregulation of cGLIS3 Circularization

**Figure 2:**
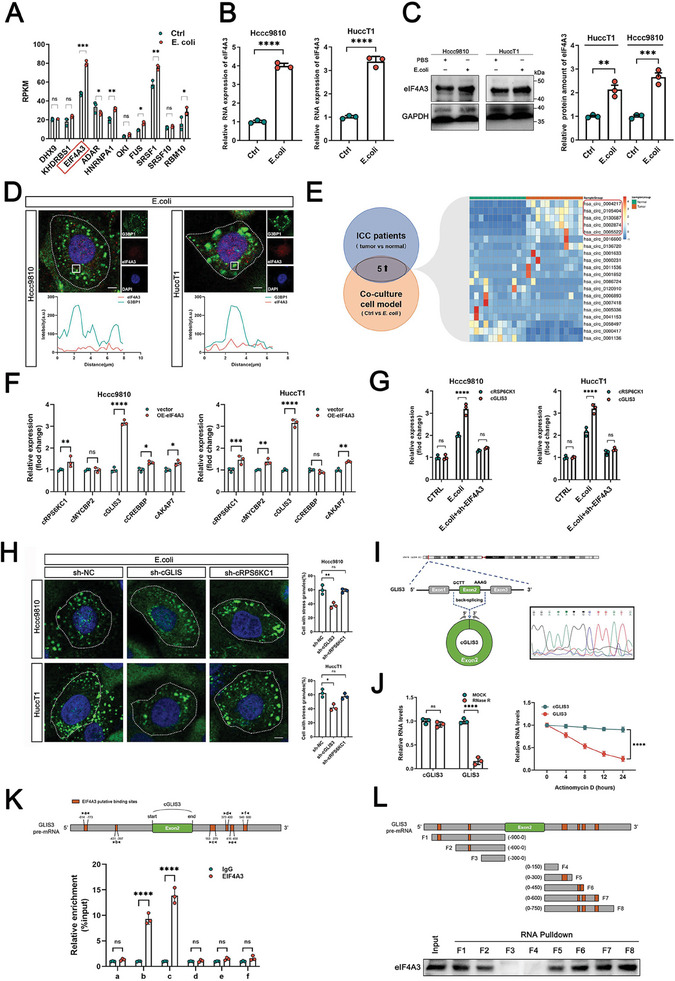
*E. coli* promotes SGs formation through the upregulation of cGLIS3 circularization. A) RNA‐seq analysis of 10 splicing factors in HuccT1 cells after *E. coli* treatment compared to the control group (PBS treatment). B) qRT‐PCR detection of relative mRNA expression of eIF4A3 in Hccc9810 and HuccT1 cells treated with *E. coli* or PBS. C) Western blotting analysis of protein expression of eIF4A3 in Hccc9810 and HuccT1 cells treated with *E. coli* or PBS. D) Immunofluorescence and the quantification of SGs (G3BP1) after *E. coli* treatment in ICC cells. E) Venn diagram shows the DEGs between 12 pairs matched ICC tissues (tumor tissue and normal tissue) and the DEGs between HuccT1 that treated with *E. coli* and PBS. Top five obtained upregulated circRNAs were further shown by using the heat‐map in ICC tumor tissue. F) qRT‐PCR detection of the expression of five obtained circRNAs in Hccc9810 and HuccT1 cells with vector/eIF4A3 overexpression. G) qRT‐PCR detection of the expression of cGLIS3 and cRSP6CK1 in Hccc9810 and HuccT1 cells treated with PBS/E*. coli/*eIF4A3 knockdown together with *E. coli*. H) Immunofluorescence of SGs (G3BP1) in Hccc9810 and HuccT1 cells treated with *E. coli* upon cGLIS3 and cRSP6KC1 knockdown. I) Schematic diagram of the genomic region of cGLIS3 and its cyclization (Left). back‐splicing junction (BSJ) of cGLIS3 identified by Sanger sequencing (Right). J) qRT‐PCR were used to analyze the expression of cGLIS3 and GLIS3 mRNA after RNase R treatment. Relative levels of cGLIS3 and GLIS3 mRNA after actinomycin D treatment were detected by qRT‐PCR at the indicated time points. K) RIP assays determined the occupancy of eIF4A3 in the intron adjacent to exon 2 of cGLIS3. L) Pulldown assays confirmed the interaction between these sites (in the introns next to exon 2 of GLIS3) and eIF4A3 protein. (A–C,F–H,J,K) For bar and line graphs, data represent mean ± SEM. (B,C,J) Unpaired two‐tailed Student's *t*‐test. (A,F,G,J,K) Two‐way ANOVA with Bonferroni's post‐hoc test (H) One‐way ANOVA with Tukey's post‐hoc test. NS indicates no significance; *p < 0.05, **p < 0.01, ***p < 0.001, and ****p < 0.0001.

Next, we examined the relationship between SGs assembly under *E. coli* stimulation and the malignant progression of ICC. The RNA‐seq results (Figure 1K) showed that the differentially expressed genes were also involved in RNA processing. Previous studies have reported that the splicing factor FUS plays an important role in regulating SGs assembly.^[^ ^18^, ^27^ ^]^ Therefore, we observed changes in the splicing factors of ICC cells after *E. coli* treatment (**Figure** **2**A). The experimental results showed that the mRNA and protein expression of the splicing factor eIF4A3 were significantly altered after treatment with *E. coli* (Figure 2B,C). Therefore, we speculate that IL6 impacts ICC cells via eIF4A3 (Figure S1E, Supporting Information).

The splicing factor eIF4A3 has been reported to promote and maintain SG stability.^[^ ^21^ ^]^ However, immunofluorescence (IF) of eIF4A3 and G3BP1 showed that they were not localized in the cytoplasm or colocalized in the condensates (Figure 2D). These results suggest that eIF4A3 is not involved in SGs formation. Considering the role of eIF4A3 in circRNA back‐splicing^[^ ^20^ ^]^ and because circRNAs are associated with SGs assembly,^[^ ^18^, ^19^, ^28^ ^]^ we speculated that the upregulation of eIF4A3 stimulated by *E. coli* might promote the formation of SGs by affecting the circularization of circRNAs. Using 12 pairs of ICC patient tissues (tumor vs normal tissue) and co‐cultured ICC cells in an indirect co‐culture model (control vs *E. coli*) to perform RNA‐seq, we screened 20 differentially expressed circRNAs. Based on their corresponding fold‐change values, we displayed their expression information through a heat map of ICC tumor tissues (Figure 2E). We focused on the top five upregulated circRNAs for further investigation. Subsequent experiments demonstrated that only cRSPKC6 and cGLIS3 showed differential changes following eIF4A3 overexpression or *E. coli* stimulation (Figure 2F,G). Only cGLIS3 knockdown reduced the increase in the number of SGs induced by *E. coli* (Figure 2H), indicating that *E. coli* may promote the formation of SGs by upregulating the expression of eIF4A3 and then increasing the circularization of cGLIS3.

cGLIS3 was generated by the circularization of exon 2 of GLIS3, which is located on chromosome 9 of the human genome (Figure 2I). Convergent and divergent primers were designed to amplify the canonical or back‐spliced isoforms of GLIS3 RNA to validate the circularization of the transcripts. The head‐to‐tail splice junction site was confirmed by polymerase chain reaction (PCR) and Sanger sequencing with divergent primers. The Ribonuclease R (RNase R) resistance analysis confirmed this result (Figure 2J). To identify eIF4A3‐binding sites in GLIS3 circRNA introns, we searched for sequences matching the potential eIF4A3 response elements near the RNA immunoprecipitation (RIP)‐enrichment regions of eIF4A3. As shown in Figure 2K, two predicted elements were located upstream of the circRNA formation splicing site, whereas the other two were located downstream. We determined the occupancy of eIF4A3 in the intron adjacent to exon 2 of cGLIS3 by RIP assay and used RT‐PCR to investigate whether eIF4A3 binds to GLIS3 pre‐mRNA (Figure 2K). Pulldown assays confirmed the interaction between these sites (in the introns next to exon 2 of GLIS3) and eIF4A3 (Figure 2L). Hence, we confirmed that eIF4A3 can bind to both sides of the cGLIS3 pre‐mRNA exon and promote circularization. *E. coli* can mediate the back‐splicing of cGLIS3 by upregulating the expression of eIF4A3, a possible key mechanism through which *E. coli* promotes SG formation.

### 2.3 *E. coli* Promotes the Progression of ICC Through cGLIS3

**Figure 3:**
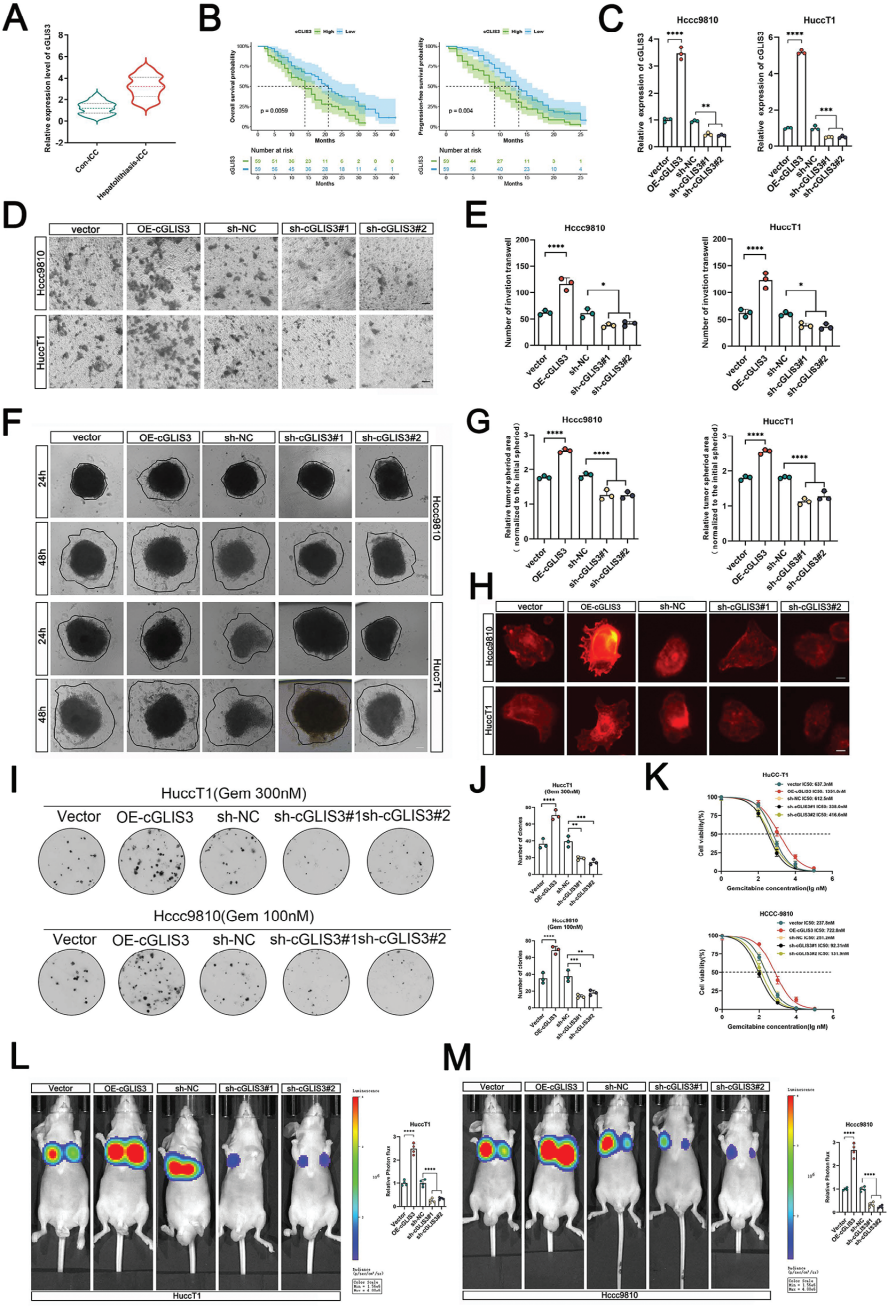
*E. coli* promotes the progression of ICC through cGLIS3. A) Expression levels of cGLIS3 were examined in ICC patients (*n* = 108). B) Kaplan–Meier survival analysis revealed a significant association between cGLIS3 expression in ICC tissues and prognosis (OS, PFS) of ICC patients. The median expression of cGLIS3 was used as the cut‐off value (*n* = 108). C) Relative RNA expression of cGLIS3 was assessed in Hccc9810 and HuccT1 cells, which were either overexpressed or knocked down using qRT‐PCR (*n* = 3). D,E) Transwell experiments were conducted to quantify the effects of cGLIS3 overexpression or knockdown, in combination with PBS or *E. coli* treatment on HuccT1 and Hccc9810 cells. Quantification of Transwell cell number was performed on the E (*n* = 3). F,G) 3D migration experiments were performed to quantify the impact of cGLIS3 overexpression or knockdown on Hccc9810 and HuccT1 cells. Quantification of 3D migration migration results was performed on the G (*n* = 3). H) Image of cytoskeleton alterations based on immunofluorescence staining of F‐actin with phalloidin (red) in Hccc9810 and HuccT1 cells with different interventions. Scale bar: 10 µm. I,J) Proliferative ability of Hccc9810 and HuccT1 cells treated with gemcitabine was investigated using colony formation assays. Representative colony formation images are shown, and the numbers of colonies are summarized on the J (*n* = 3). K) IC50 experiments were conducted on HuccT1 and Hccc9810 cells, which were overexpressing or knocked down for cGLIS3, and treated with PBS or *E. coli* treatment. L) HuccT1 cells with overexpressing cGLIS3, knockdown cGLIS3 and the control were injected into nude mice via the tail vein. Representative images and statistical analysis of in vivo lung bioluminescence in the lung metastasis model (*n* = 4). M) Hccc9810 cells with overexpressing cGLIS3, knockdown cGLIS3 and the control were injected into nude mice via the tail vein. Representative images and statistical analysis of in vivo lung bioluminescence in the lung metastasis model (*n* = 4). (C,E,G,J–M) For bar and line graphs, data represent mean ± SEM. (C,E,G,J,L,M) One‐way ANOVA with Tukey's post‐hoc test (K) Two‐way ANOVA with Bonferroni's post‐hoc test NS: no significant difference. **p < 0.01, ***p < 0.001, and ****p < 0.0001.

Next, we found that cGLIS3 was highly expressed in ICC tissues compared to that in normal tissues (Figure S2A, Supporting Information). Notably, the expression of cGLIS3 was higher in patients with hepatolithiasis‐related ICC than ICC patients with ICC without hepatolithiasis (Con‐ICC) (**Figure** **3**A). ICC patients with high cGLIS3‐expression levels showed poorer overall survival (OS) and disease‐free survival (PFS) (Figure 3B).

To investigate the potential role of cGLIS3 as a key downstream molecule promoted by *E. coli* in ICC progression, we assessed its functional role of cGLIS3 in ICC by using clinical samples and in vitro cell models. We generated stable HuccT1 and Hccc9810 cell lines that either overexpressed or had knocked down cGLIS3. We verified the construction of these stable cell lines using qPCR (Figure 3C). Functionally, the overexpression of cGLIS3 further enhanced the migration ability (Figure 3D–H) and gemcitabine resistance (Figure 3I–K) of ICC cells pretreated with *E. coli*, whereas its knockdown yielded the opposite effects. In vivo experiments also confirmed that ICC cells overexpressing cGLIS3 had a greater metastatic capacity (Figure 3L,M). Based on these results, we can conclude that *E. coli* promotes SGs formation through the upregulation of cGLIS3, ultimately affecting the migration and drug resistance of ICC.

### 2.4 cGLIS3 Forms a Complex with hnRNPA1 and G3BP1

**Figure 4:**
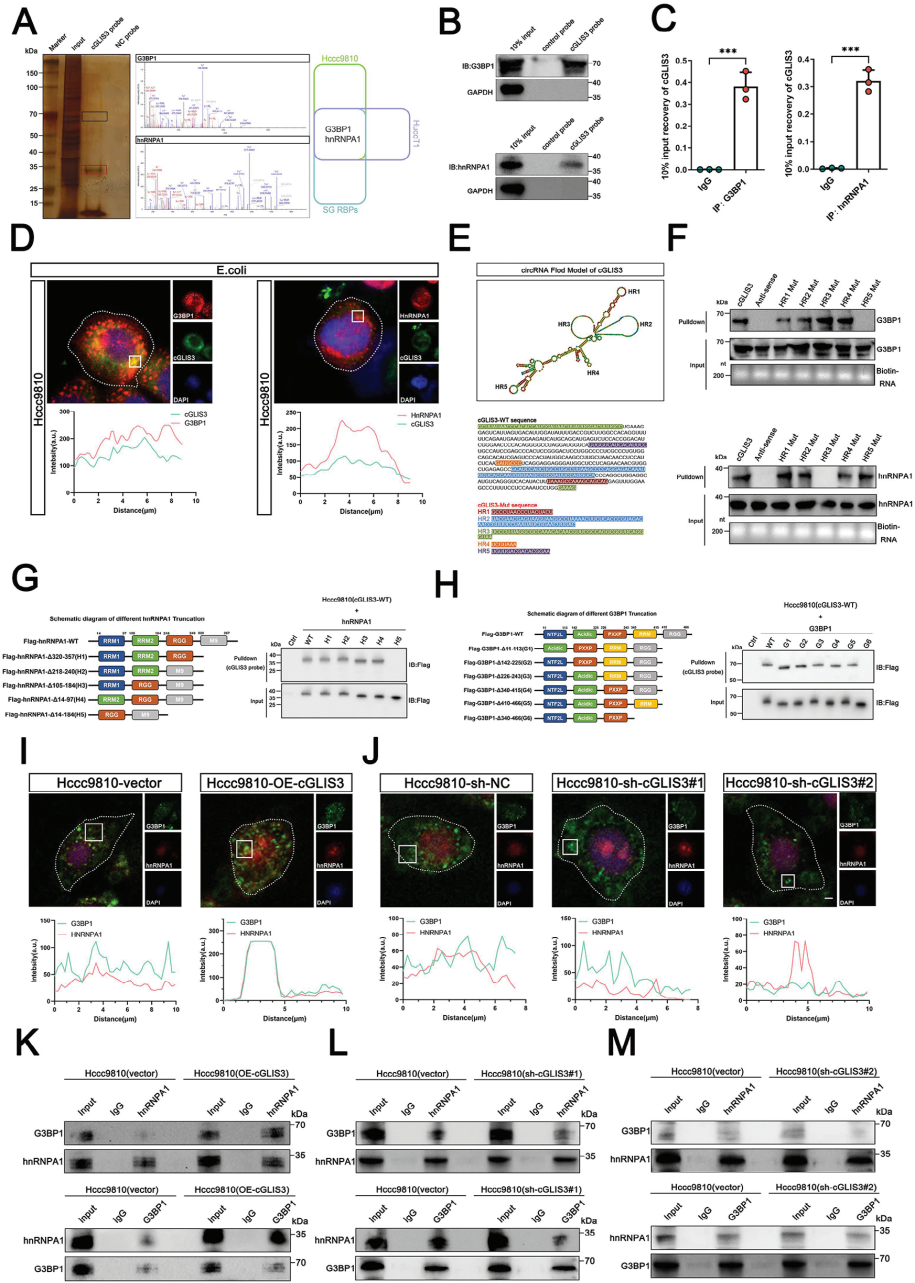
cGLIS3 forms a complex with hnRNPA1 and G3BP1. A) cGLIS3‐specific probe‐bound RBP by silver staining and mass spectrometry. B) Binding of hnRNPA1/G3BP1 and cGLIS3, as shown by RNA‐pulldown experiment. C) Binding of hnRNPA1/G3BP1 and cGLIS3, as shown by RIP experiment. D) Upper: Immunofluorescence analysis of hnRNPA1 and G3BP1 in Hccc9810 cells, nuclei are stained with DAPI. Scale bar, 10 µm. Below: Curves of fluorescence intensities of the position marked by a white square in the merged image. E) Upper: Mutations to abrogate loop structures of cGLIS3. Below: The sequences of cGLIS3‐WT, cGLIS3‐HR1‐mut, cGLIS3‐HR2‐mut, cGLIS3‐HR3‐mut, cGLIS3‐HR4‐mut, and cGLIS3‐HR5‐mut. F) RNA pulldown assay was performed using cGLIS3‐WT, cGLIS3‐HR1‐mut, cGLIS3‐HR2‐mut, cGLIS3‐HR3‐mut, cGLIS3‐HR4‐mut, and cGLIS3‐HR5‐mut. G) Left: Diagram of the wild‐type hnRNPA1 and different truncations. Right: After co‐transfecting cGLIS3‐WT and the full‐length or different hnRNPA1 truncations with Flag, RNA pull down assays performed using cGLIS3 probe. H) Left: Diagram of the wild‐type G3BP1 and different truncations. Right: After co‐transfecting cGLIS3‐WT and the full‐length or different G3BP1 truncations with Flag, RNA pull down assays performed using cGLIS3 probe. I) Upper: Immunofluorescence analysis of hnRNPA1 and G3BP1 in Hccc9810 cells upon the overexpression of cGLIS3. Nuclei are stained with DAPI. Scale bar, 10 µm. Below: Curves of fluorescence intensities of the position marked by a white square in the merged image. J) Upper: Immunofluorescence analysis of hnRNPA1 and G3BP1 in Hccc9810 cells upon the knockdown of cGLIS3. Nuclei are stained with DAPI. Scale bar, 10 µm. Below: Curves of fluorescence intensities of the position marked by a white square in the merged image. K–M) Binding effect of hnRNPA1 and G3BP1 after overexpression or knockdown of cGLIS3, as shown by COIP experiment in Hccc9810. (C) For bar and line graphs, data represent the mean ± SEM. (C) Unpaired two‐tailed Student's *t*‐test. NS: no significant difference. ***p < 0.001.

RNA‐binding proteins (RBPs) with a prion‐like domain (PrLD) undergo phase transition into functional liquids and play an important role in SG formation.^[^ ^28^ ^]^ CircRNAs usually function through a competing endogenous RNA (ceRNA) mechanism or by binding to RBP. However, our results showed that cGLIS3 does not bind to AGO2 (Argonaute2) (Figure S2B, Supporting Information), suggesting that cGLIS3 functions mainly by binding to RBP. RBP binding is a major activity of circRNAs in vivo. For example, circFoxo3 promotes cell senescence by binding to age‐related proteins. To investigate the regulatory mode and molecular mechanism of cGLIS3, we used biotin‐labeled cGLIS3 sense and antisense probes for RNA pull‐downs. In addition, we conducted a mass spectrometry analysis of Hccc9810 and HuccT1 cells. Enrichment of both hnRNPA1 and G3BP1 was observed in the cGLIS3 pull‐down precipitates of Hccc9810 and HuccT1 cells (**Figure** **4**A). RNA pull‐down (Figure 4B) and RIP experiments (Figure 4C) also confirmed the binding between hnRNPA1/G3BP1 and cGLIS3. Immunofluorescence assays showed that hnRNPA1 and G3BP1 also colocalized with cGLIS3 in SGs formed by *E. coli* stimulation (Figure 4D). We predicted the loop structure of cGLIS3 using a bioinformatics tool (RNAfold WebServer; http://rna.tbi.univie.ac.a) and constructed different cGLIS3 mutations to abrogate the loop structures (Figure 4E). To identify the loops (HR1, HR2, HR3, HR4, and HR5) indispensable for the association of cGLIS3 and hnRNPA1/G3BP1, we performed an RNA pull‐down assay using the biotin‐labeled mutant cGLIS3 (Figure 4F). We observed that the HR5 mutation in cGLIS3 abolished its association with hnRNPA1, whereas the HR3 mutation abolished its association with G3BP1. We constructed different truncations of hnRNPA1 (Figure 4G) and G3BP1 (Figure 4H). RNA pull‐down and RIP assays showed that the 14–184 regions of hnRNPA1 and the 255–419 regions of G3BP1 were crucial for interaction with circGLIS3(Figure 4G,H; Figure S2F,G, Supporting Information). Importantly, the immunofluorescence co‐localization of hnRNPA1 and G3BP1 was enhanced after the overexpression of cGLIS3 and reduced after the knockdown of cGLIS3 (Figure 4I,J). Co‐immunoprecipitation (COIP) experiments further validated that the overexpression of cGLIS3 enhanced the binding between hnRNPA1 and G3BP1, whereas its knockdown significantly weakened this binding (Figure 4K–M). These results indicated that binding between cGLIS3 and hnRNPA1/G3BP1 occurs on HR3 and HR5 of cGLIS3.

### 2.5 cGLIS3/hnRNPA1/G3BP1 Complex Promotes ICC Progression via SGs Formation

**Figure 5:**
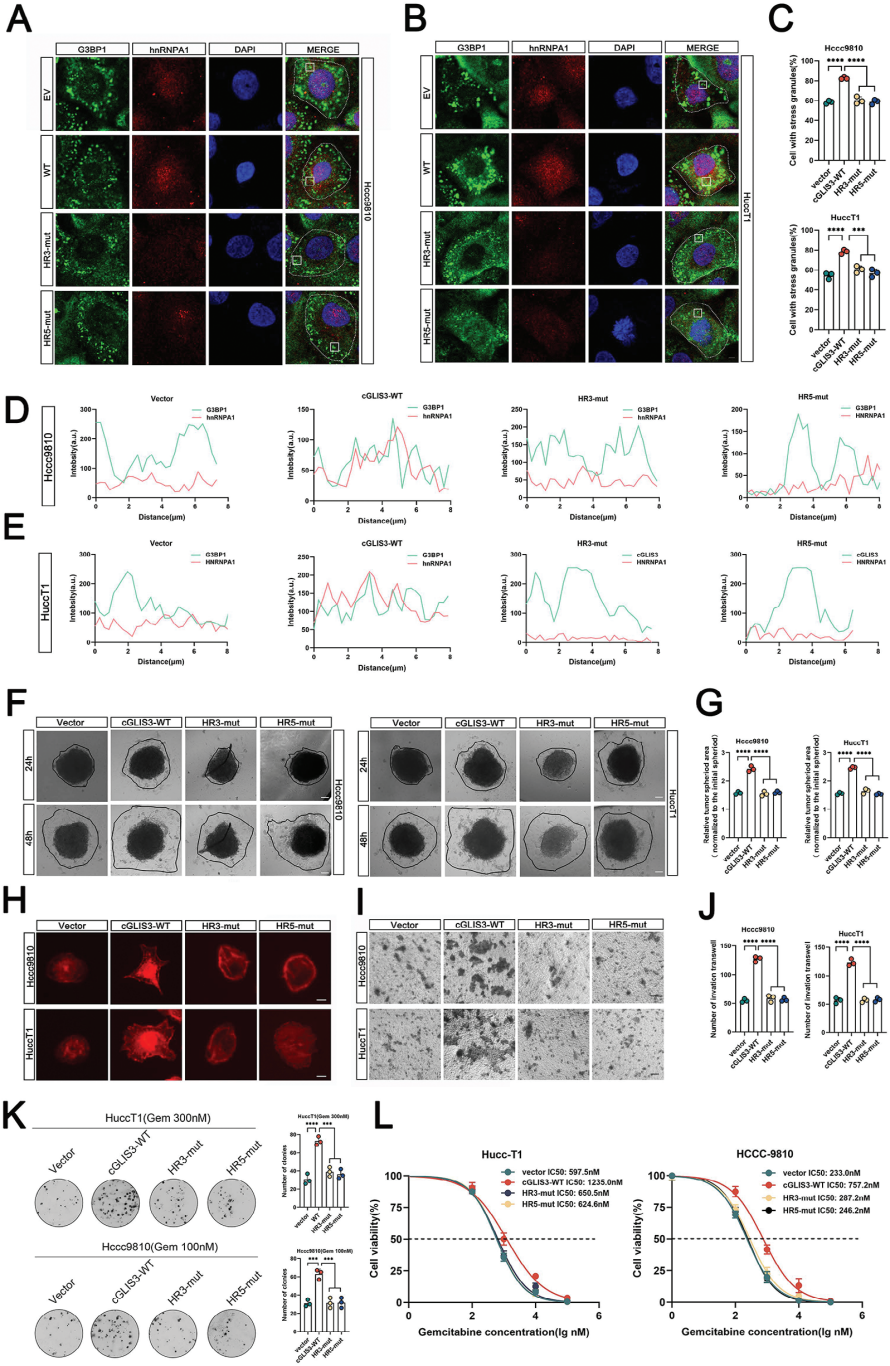
cGLIS3/hnRNPA1/G3BP1 complex promotes ICC progression via SGs formation. A,B) Effect of cGLIS3‐WT, cGLIS3‐HR3‐mut, and cGLIS3‐HR5‐mut on the increased formation of SGs observed by immunofluorescence experiment in Hccc9810 (A) and HuccT1 (B) cells; C) Display of quantification results of SG (G3BP1) immunofluorescence in Hccc9810 (Upper) and HuccT1 (Below) cells (*n* = 3). D) Curves of fluorescence intensities of the position marked by a white square in the merged image of Hccc9810 cell. E) Curves of fluorescence intensities of the position marked by a white square in the merged image of HuccT1 cells. F) Effect of cGLIS3‐WT, cGLIS3‐HR3‐mut and cGLIS3‐HR5‐mut on the migration ability, as observed through 3D migration experiments in Hccc9810 and HuccT1 cells. G) Display of quantification results of 3D migration experiments in Hccc9810 (Left) and HuccT1 (Right) cells (*n* = 3). H) Effect of cGLIS3‐WT, cGLIS3‐HR3‐mut, and cGLIS3‐HR5‐mut on cytoskeleton alterations by immunofluorescence staining of F‐actin with phalloidin (red) in Hccc9810 and HuccT1 cells . Scale bar: 10 µm. I,J) Effect of cGLIS3‐WT,cGLIS3‐HR3‐mut and cGLIS3‐HR5‐mut on the migration ability, as observed through Transwell experiments (I). Quantification of invasion number was performed on the right image (J) (*n* = 3). K) Proliferative ability of stably transfected cGLIS3‐WT, cGLIS3‐HR3‐mut and cGLIS3‐HR5‐mut Hccc9810 and HuccT1 cells treated with gemcitabine was investigated using colony formation assays. Representative colony formation images are shown, and the numbers of colonies are summarized on the right (*n* = 3). L) Effect of cGLIS3‐WT, cGLIS3‐HR3‐mut, and cGLIS3‐HR5‐mut on gemcitabine resistance, as observed through IC50 experiment. (C,G,J–L) For bar and line graphs, data represent mean ± SEM. (C,G,J,K) One‐way ANOVA with Tukey's post‐hoc test (L) Two‐way ANOVA with Bonferroni's post‐hoc test. NS, no significance; ***p < 0.001 and ****p < 0.0001.

To further investigate the role of the cGLIS3/hnRNPA1/G3BP1 complex and SGs in ICC, we carried out subsequent experiments by constructing cGLIS3‐HR3‐MUT‐ and cGLIS3‐HR5‐MUT‐overexpressed cells. cGLIS3‐HR3 and cGLIS3‐HR5 have been shown to occur in the region between cGLIS3 and hnRNPA1/G3BP1 (Figure 4E,F). Interestingly, our results demonstrate that cGLIS3‐HR3‐MUT and cGLIS3‐HR5‐MUT do not promote SGs formation. In addition, they did not stabilize the binding between hnRNPA1 and G3BP1 in the SGs (**Figure** **5**A–C). This was considerably different from the role of cGLIS3‐WT. Moreover, the immunofluorescence co‐localization of hnRNPA1 and G3BP1 was reduced upon HR3‐mut and HR5‐mut treatment of cGLIS3 (Figure 5D,E). Importantly, with the loss of cGLIS3‐HR3‐MUT and cGLIS3‐HR5‐MUT roles in SGs formation, their roles in promoting ICC progression were also lost. Our results showed that overexpression of cGLIS3‐HR3‐MUT and cGLIS3‐HR5‐MUT did not enhance the migration ability (Figure 5F–H) and drug resistance (Figure 5I–L) of ICC cells. These results suggest that cGLIS3 promotes SGs formation and ICC progression by binding to hnRNPA1 and G3BP1.

### 2.6 cGLIS3‐Mediated SGs Formation Inhibits the Ubiquitination of hnRNPA1 and G3BP1

**Figure 6:**
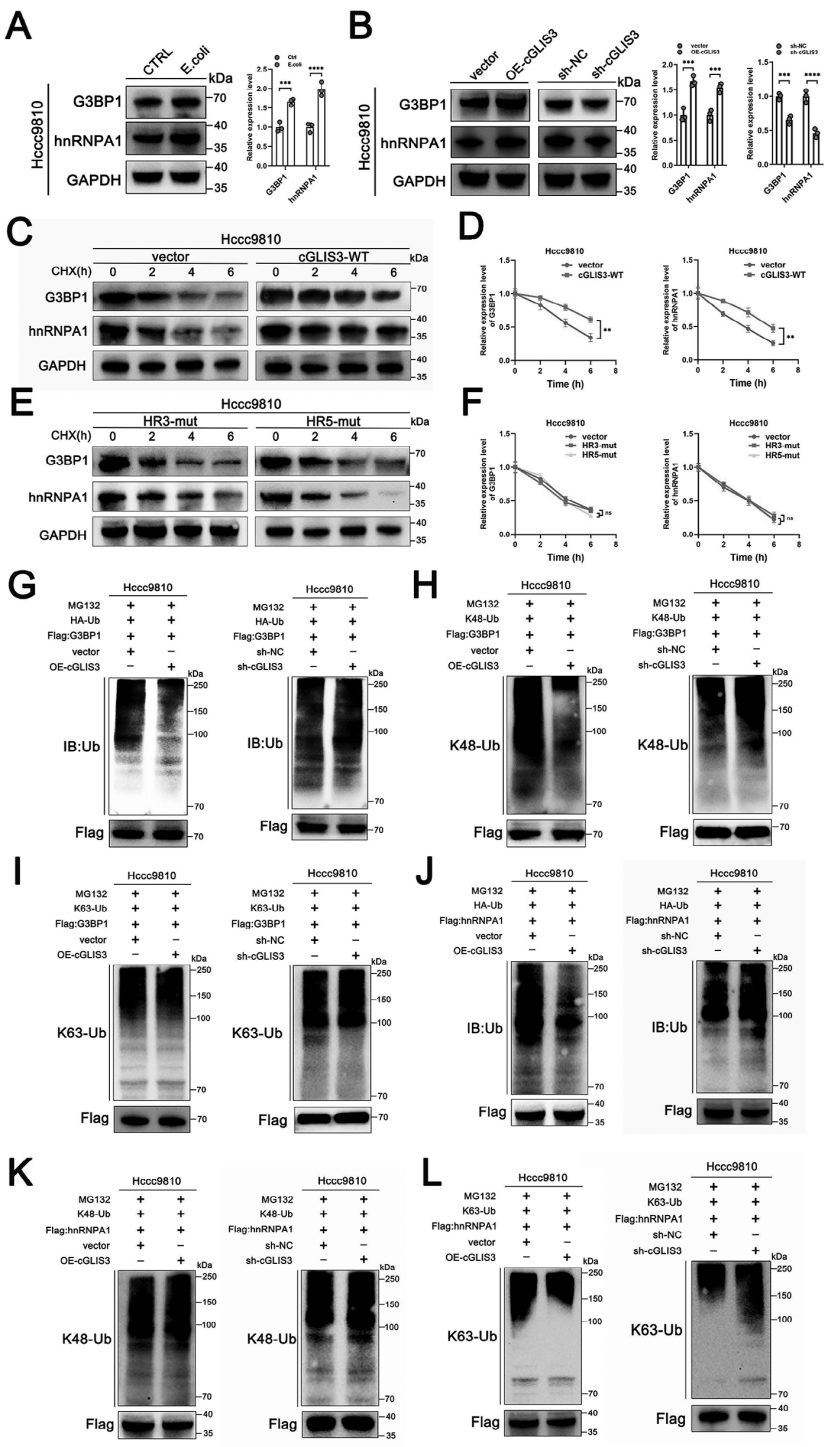
cGLIS3‐mediated SGs formation inhibits the ubiquitination of hnRNPA1 and G3BP1. A) Western blot analysis of G3BP1 and hnRNPA1 in Hccc9810 cells pretreated with *E. coli* or PBS. B) Western blot analysis of G3BP1 and hnRNPA1 upon cGLIS3 overexpression or knockdown in Hccc9810 cells. C,D) Western blot analysis of the half‐life of G3BP1 and hnRNPA1 in vector or cGLIS3‐WT Hccc9810 cells treated with cGLIS3‐WTand CHX (400 µg mL^−1^). Quantification of Western blot analysis of the half‐life of G3BP1 and hnRNPA1 is shown on the right image. E,F) Western blot analysis of the half‐life of G3BP1 and hnRNPA1 in cGLIS3‐HR3‐mut and cGLIS3‐HR5‐mut Hccc9810 cells treated with CHX (400 µg mL^−1^). Quantification of Western blot analysis of the half‐life of G3BP1 and hnRNPA1 is shown on the right image. G–I) Ubiquitination of exogenous G3BP1 in Hccc9810 cells transfected with indicated plasmids with MG132 (20 µM) pretreatment. Western blot analysis was performed using Ub, K48‐Ub, K63‐Ub and Flag antibodies. J–L) Ubiquitination of exogenous hnRNPA1 in Hccc9810 cells transfected with indicated plasmids with MG132 (20 µM) pretreatment. Western blotting was performed using Ub, K48‐Ub, K63‐Ub, and Flag antibodies. (A,B,D,F): Bar and line graphs; data represent the mean ± SEM. (A,B) One‐way ANOVA with Tukey's post‐hoc test (D,F) Two‐way ANOVA with Bonferroni's post hoc test. NS, no significance; ***p < 0.001 and ****p < 0.0001.

We further investigated the effect of cGLIS3‐forming SGs on protein expression of the cGLIS3/hnRNPA1/G3BP1 complex. Our results demonstrate the upregulation of hnRNPA1 and G3BP1 protein expression following *E. coli* stimulation (**Figure** **6**A). We investigated the effects of cGLIS3 on hnRNPA1 and G3BP1 expression. Although cGLIS3 had no regulatory effect on hnRNPA1 and G3BP1 at the transcriptional level (Figure S2C,D, Supporting Information), it did regulate protein expression at the post‐transcriptional level. We observed that overexpression of cGLIS3 increased hnRNPA1 and G3BP1 protein levels, whereas its silencing decreased these levels (Figure 6B). After treatment with the protein synthesis inhibitor cycloheximide (CHX), overexpression of cGLIS3 increased the half‐life of hnRNPA1 and G3BP1, whereas its mutation had no impact on the degradation of hnRNPA1 and G3BP1 in Hccc9810 cells (Figure 6C–F). Therefore, we hypothesized that cGLIS3 plays a role in regulating hnRNPA1 and G3BP1 protein stability. ICC cells have two major protein degradation pathways: ubiquitination and autophagy. Moreover, it has been reported that hnRNPA1‐mediated liquid–liquid phase separation (LLPS) contributes to the assembly and liquid‐like characteristics of SGs and that the ubiquitination of G3BP1 mediates SGs disassembly.^[^ ^18^, ^29^ ^]^ We conducted the CHX treatment, and treated Hccc9810 cells with MG132, a proteasome inhibitor, to investigate the role of cGLIS3 in the regulation of hnRNPA1 and G3BP1 stability. The results showed that cGLIS3 regulates the stability of hnRNPA1 and G3BP1 through the ubiquitination pathway and subsequently modulates their protein expression.

Next, we explored whether cGLIS3 is involved in regulating hnRNPA1 and G3BP1 ubiquitination. We performed exogenous transfection with HA‐Ub, Flag‐G3BP1, Flag‐hnRNPA1, and cGLIS3 probes. Treatment with the proteasome inhibitor, MG132, significantly decreased the ubiquitination of hnRNPA1 and G3BP1 in cGLIS3‐overexpressing cells. Similarly, cGLIS3 knockdown greatly increased the accumulation of ubiquitinated Flag‐hnRNPA1 and Flag‐G3BP1 in Hccc9810 cells (Figure 6G,J). We investigated the effect of cGLIS3 on K48 or K63 polyubiquitination of hnRNPA1 and G3BP1. Subsequently, exogenous transfection with K48‐Ub, Flag‐G3BP1, and cGLIS3 was performed. Treatment with MG132 significantly decreased the K48 ubiquitination of hnRNPA1 and G3BP1 in cGLIS3‐expressing cells (Figure 6H,K). Likewise, the knockdown of cGLIS3 significantly increased the accumulation of K48‐ubiquitinated Flag‐G3BP1 in Hccc9810 cells. Next, we performed exogenous transfection with K63‐Ub, Flag‐G3BP1, and cGLIS3. MG132 treatment did not cause any significant difference in K63 ubiquitination of exogenous hnRNPA1 and G3BP1 between the high cGLIS3‐expression group and control groups (Figure 6I,L). Hccc9810 cells lost the potential to regulate K63 ubiquitination of Flag‐hnRNPA1 and Flag‐G3BP1 owing to the knockdown of cGLIS3. The level of K63‐related poly‐ubiquitination did not differ significantly between the two groups. Our results confirmed that cGLIS3 stabilizes hnRNPA1 and G3BP1 by inhibiting K48‐related polyubiquitination, thereby stabilizing SGs and promoting ICC progression.

### 2.7 SGs Promote the Progression of ICC by Activating the NF‐κB Signaling Pathway

**Figure 7:**
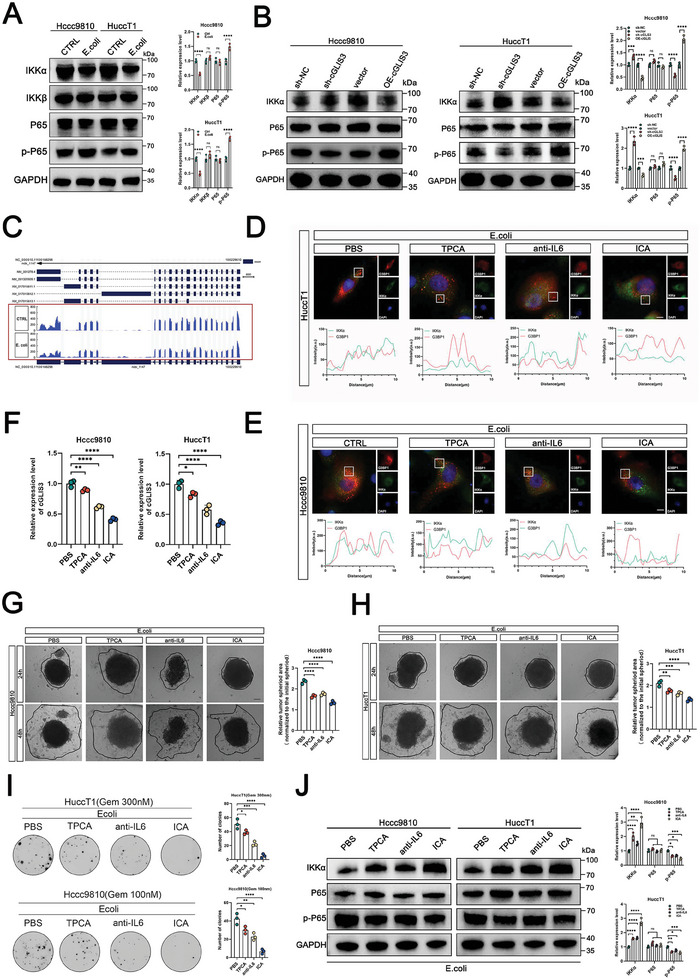
SGs promote the progression of ICC by activating the NF‐kB signaling pathway. A) Changes in NF‐κB pathway after *E. coli* stimulation, as observed by Western blot experiment. Quantification of Western blot analysis is shown on the right (*n* = 3). B) Changes in the NF‐κB pathway when cGLIS3 is overexpressed or knocked down in Western Blot experiment in the Hccc9810 and HuccT1 cells. Quantification of Western blot analysis is shown on the right (*n* = 3). C) Effect of pretreated with *E. coli* or PBS on IKKα mRNA translation level of the ICC cells based on RNC sequencing. D) Upper: Immunofluorescence analysis of IKKα mRNA and G3BP1 in HuccT1 cells pretreated with *E. coli* upon PBS, TPCA, anti‐IL6, and ICA. Nuclei are stained with DAPI. Scale bar, 10 µm. Below: Curves of fluorescence intensities of the position marked by a white square in the merged image. E) Upper: Immunofluorescence analysis of IKKα mRNA and G3BP1 in Hccc9810 cells pretreated with *E. coli* upon PBS, TPCA, anti‐IL6, and ICA. Nuclei are stained with DAPI. Scale bar, 10 µm. Below: Curves of fluorescence intensities of the position marked by a white square in the merged image. F) Effect of PBS, TPCA, anti‐IL6, and ICA changes on cGLIS3 expression level in Hccc9810 and HuccT1 cells, as shown by the qPCR experiment (*n* = 3). G,H) Effect of PBS, TPCA, anti‐IL6, and ICA on the metastatic ability of Hccc9810 and HuccT1 cells after pretreatment with *E. coli*, as demonstrated by a 3D migration experiment. Quantification of migration is shown on the right image (*n* = 3). I) Proliferative ability of gemcitabine‐treated Hccc9810 and HuccT1 cells with different interventions was investigated using colony formation assays. Representative colony formation images are shown. Numbers of colonies are summarized on the right (*n* = 3). J) Effect of ICA on the NF‐κB pathway of Hccc9810 and HuccT1cells after pretreatment with *E. coli*, as demonstrated by Western blot analysis. Quantification of Western blot is shown on the right image (*n* = 3). (A,B,F,G–J) For bar and line graphs, data represent mean ± SEM. (F,G–I) One‐way ANOVA with Tukey's post‐hoc test (A,B,J) Two‐way ANOVA with Bonferroni's post‐hoc test. NS indicates no significance; *p < 0.05, **p < 0.01, ***p < 0.001, and ****p < 0.0001.

Next, we elucidated the specific mechanisms by which circRNAs promote ICC progression via the SGs. By combining our different sequencing results (Figure 1H), we observed a correlation between ICC patients with hepatolithiasis and the activation of the NF‐κB pathway (**Figure** **7**A). We examined changes in the NF‐κB pathway through Western blot analysis and observed that the expression of IKKα protein was upregulated in Hccc9810 and HuccT1 cells with silenced cGLIS3, while it was downregulated in these cells with overexpressed cGLIS3 (Figure 7B). Our results showed that *E. coli* had no regulatory effect on IKKα at the transcriptional level (Figure S2E, Supporting Information). Moreover, we conducted the RNC sequencing analysis on the ICC cells treated with *E. coli* or PBS, and it indicated that SGs may activate the NF‐κB pathway and primarily influence the translation of IKKα mRNA (Figure 7C). We further used TPCA (NF‐κB inhibitor), anti‐IL6, and ICA (drugs with simultaneous inhibitory effects on NF–κB and IL6)^[^ ^30^, ^31^ ^]^ to observe their effects on SGs formation. Immunofluorescence experiments showed that although TPCA and anti‐IL6 had some inhibitory effect on the binding of IKKα and G3BP1, only ICA demonstrated a striking effect (Figure 7D,E). Additionally, ICA caused a greater decrease in cGLIS3 levels than TPCA and anti‐IL6 (Figure 7F). Our results also showed that the enhanced migration capability (Figure 7G,H) and drug resistance (Figure 7I) of Hccc9810 cells co‐cultured with *E. coli* or overexpressing cGLIS3 were greatly restored after the addition of ICA. This effect was greater than that observed in the TPCA and anti‐IL6 groups. When cGLIS3 was overexpressed, SGs sequestered the inhibitory factor IKKα mRNA of the NF‐κB pathway, leading to sustained activation of NF‐κB (Figure 7J).

### 2.8 ICA in Combination with GP Effectively Inhibits the Progression of ICC

**Figure 8:**
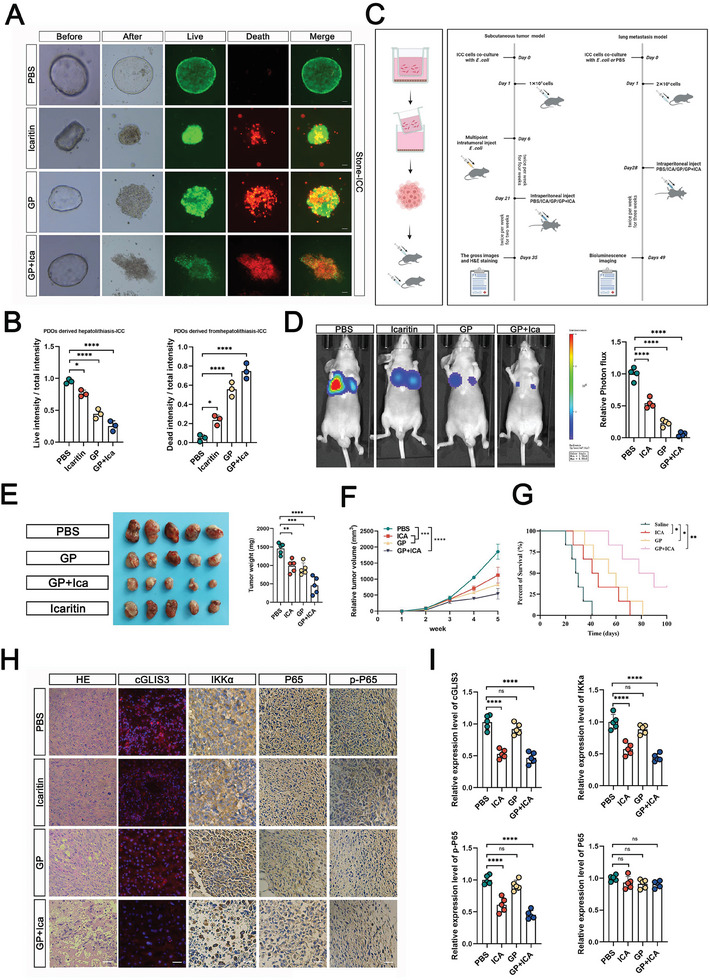
ICA in combination with GP effectively inhibits the progression of ICC. A,B) Effect of ICA combined with GP on cells observed through an PDOs model. Quantification is shown in B. Live/Dead cell viability assay showing live cells stained with Calcein‐AM (green) and dead cells with EthD‐1 (red) (*n* = 3). C) Schematic diagram of subcutaneous tumor and lung metastasis model, respectively (*n* = 4). D) Representative images and statistical analysis of in vivo lung bioluminescence in the lung metastasis model with indicated treatments. E,F) Subcutaneous tumor formation in mice after different interventions, respectively (*n* = 5). Tumors were extracted and weighed after mice were sacrificed. Tumor size was measured at the indicated time points. G) Survival time of the lung metastasis mouse model after different interventions. H,I) Representative images of H&E staining, cGLIS3 (FISH) staining, IKKα (IHC) staining, p‐P65 (IHC) staining, and P65 (IHC) staining in the orthotopic tumors with indicated treatments. Quantification of IHC source is shown in the right image, respectively (*n* = 5). Scale bar: 50 µm. (B,D,F,G,I) For bar and line graphs, data represent mean ± SEM. (B,D,F,G,I) One‐way ANOVA with Tukey's post hoc test NS indicates no significance; *p < 0.05, **p < 0.01, ***p < 0.001, and ****p < 0.0001.

We first constructed an organoid model using cancer cells obtained from patients with ICC hepatolithiasis to investigate the in vivo role of cGLIS3. This model was used to validate the effects of ICA (**Figure** **8**A,B). We further validated the effects of the ICA in an in vivo nude mouse subcutaneous model subjected to different treatments. Four weeks after tumor cell injection, we administered different intervention treatments (PBS, gemcitabine plus cisplatin (GP, ICA, and GP + ICA) and further assessed the tumor size for 3 weeks (Figure 8C). Similar results were observed in the lung metastasis model. The GP‐ICA group exhibited the highest antitumor efficacy (Figure 8D–G). In addition, we found that GP‐ICA effectively silenced cGLIS3, increased the degree of IKKα levels, and reduced the NF‐κB pathway in the subcutaneous model (Figure 8H,I). Our findings provide a novel molecular mechanism for the progression of ICC in patients with hepatolithiasis and demonstrate the crucial role of ICA in this specific patient population.

## 3 Discussion

**Figure 9:**
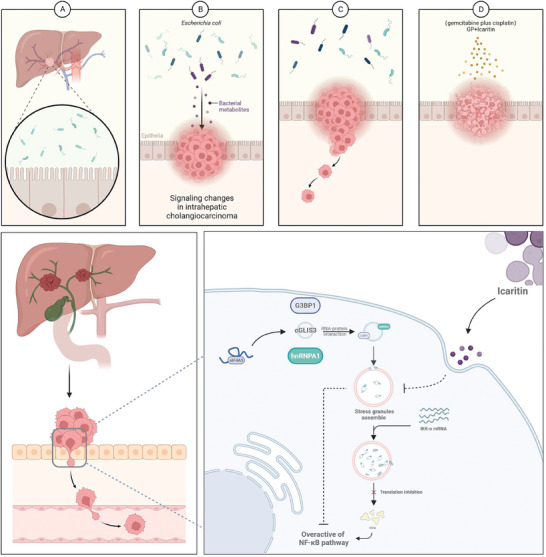
Graphical illustration of ICA targeting the Ecoli‐cGLIS3 axis to inhibit SGs formation.

ICC with hepatolithiasis has poor prognosis because of the presence of bacteria in the biliary tract. *E. coli* is the predominant bacterium found in hepatolithiasis–ICC patients.^[^ ^32^, ^33^ ^]^ Bacteria have been shown to play a crucial role in promoting the aggressive progression of cancer, leading to highly malignant cell behavior and poor response to chemotherapy.^[^ ^34^, ^35^, ^36^ ^]^ Previous studies by Chai et al. have explored the microbial characteristics within ICC, confirming the potential role of microbiota in cancer treatment.^[^ ^6^ ^]^ In this study, we focused on the potential pro‐cancerous effects of *E. coli* on the bile microenvironment. In line with previous studies, we confirmed that *E. coli* was the predominant bacterium in the bile of patients with ICC and hepatolithiasis. Although *E. coli* has been reported to promote the progression of colon cancer,^[^ ^9^ ^]^ the relationship between *E. coli* and ICC progression remains is still not fully understood. Our findings support that *E. coli* increases the malignancy of ICC by upregulating the expression of splicing factor eIF4A3, thereby promoting the assembly of SGs and leading to sustained activation of the NF‐κB pathway. Furthermore, we identified the role of a novel NF‐κB inhibitor named ICA through in vitro/in vivo and PDOs models, demonstrating that ICA plays a crucial role in inhibiting SGs formation and increasing the sensitivity to GP chemotherapy in ICC(**Figure** **9**).

SGs are cytoplasmic membrane‐less RNP aggregates that form in response to various environmental stressors such as heat shock, oxidative stress, osmotic stress, or nutrient starvation.^[^ ^37^, ^38^ ^]^ Previous studies have reported that SGs influence the malignant behavior of tumors, particularly their invasion, migration, and drug resistance.^[^ ^12^, ^16^ ^]^ Our research showed that ICC stimulated with *E. coli* exhibited enhanced migratory ability and chemoresistance. A significant increase in the number of SGs was observed. Interestingly, when SGs were reduced, a corresponding decrease in the malignant behavior of the tumor was observed. This finding regarding the relationship between SGs, tumor metastasis, and drug resistance is consistent with those of previous studies. Our results demonstrate, for the first time, a correlation between *E. coli*‐promoted ICC progression and SGs formation, providing further insights into the potential factors underlying *E. coli*‐induced ICC progression and SG formation.

Previous studies have reported that pathogenic bacteria can stimulate SGs formation via the action of exotoxins.^[^ ^13^ ^]^ We found that *E. coli* stimulation led to the upregulation of the splicing factor eIF4A3, which is involved in the formation of SGs as it interacts with circRNA circularization.^[^ ^21^, ^24^ ^]^ However, eIF4A3 is not the main component of SGs. Therefore, we investigated whether increased circRNA levels generated by eIF4A3 splicing were involved in SGs formation. We report for the first time that the circRNA cGLIS3, generated through eIF4A3 splicing, is associated with SGs assembly. By analyzing clinical specimens and conducting in vitro and in vivo experiments, we found that elevated cGLIS3 expression correlated with poor prognosis in patients with ICC. Furthermore, cGLIS3 promotes the invasion, migration, and chemoresistance of ICC cells. This functional role in promoting malignant behavior is closely associated with the ability of cGLIS3 to stimulate SG formation. Our findings confirm that *E. coli*‐induced ICC progression requires cGLIS3 for SGs formation, which further elucidates the role of circRNAs in ICC.

Next, we explored the mechanisms by which circRNAs promote SG formation. An important mechanism of circRNAs involves their binding to RBPs. Our silver staining results revealed that cGLIS3 could bind to hnRNPA1 and G3BP1, which are typical heterogeneous RNPs (hnRNPs) composed of two folded RNA‐recognition motifs and a low‐complexity sequence domain.^[^ ^39^ ^]^ The former occupies the N terminus of the protein, whereas the latter occupies the C terminus. HnRNPA1 is one of a component of SGs. We found that cGLIS3 promoted SG production and required hnRNPA1 expression. In addition, COIP results confirmed that cGLIS3 promotes the formation of a more stable complex between hnRNPA1 and G3BP1. Knockdown of cGLIS3 affects the combination of hnRNPA1 and G3BP1, which is an important factor in cGLIS3‐mediated SGs generation. The components of RNA‐binding proteins assembled by the core (e.g., G3BP1) and the increased combination of SGs assembly element proteins (e.g., hnRNPA1) are important for the formation of stable SGs structures.^[^ ^37^ ^]^ Our results demonstrated that cGLIS3 maintains SG stability by inhibiting the ubiquitination of hnRNPA1 and G3BP1. This result is similar to that reported previously, according to which circRNAs mediate protein ubiquitination.^[^ ^18^, ^29^, ^40^ ^]^ Although there could be complex ubiquitination mechanisms may exist, we have not yet identified a clear mechanism mediated by cGLIS3. Our study indicated that cGLIS3‐HR3∖HR5‐mut was unable to promote SG formation or reduce the ubiquitination levels of hnRNPA1 and G3BP1. Hence, there was no increase in the malignant behavior of ICC cells. Therefore, SG formation is essential for cGLIS3 to contribute to ICC progression.

SG formation can often cause crosstalk among intracellular signaling pathways by condensing specific target proteins or mRNAs. For example, circVAMP3 inhibits the expression of the Myc proto‐oncogene protein by inhibiting the translation of c‐Myc through SGs.^[^ ^19^ ^]^ *E. coli*‐stimulated ICC cells show abnormal activation of the NF‐κB pathway, which plays an important regulatory role in the metastasis and drug resistance of malignant tumors, including ICC.^[^ ^22^ ^]^ Note that cGLIS3‐mediated SGs cause the continuous activation of the NF‐κB pathway. The SGs blocked the mRNA of IKKα during the formation of the assembly. This will cause continuous activation of the NF‐κB pathway signal, thereby promoting ICC invasion, migration, and chemotherapy resistance. This mechanism is different from the previous regulatory mechanism of the NF‐κB pathway, revealing a new way for *E. coli* to stimulate the activation of the NF‐κB pathway in ICC cells and tumor progression.

Previous studies have suggested that *E. coli* may require cytokines to promote cancer. Our results showed that the upregulation of IL6 was particularly abnormal during *E. coli* infection. IL6 is a pleiotropic cytokine produced in response to stress and inflammatory stimuli and plays an important role in tumorigenesis and cancer progression. IL6 promotes eIF4A3 expression. According to our results, the increase in SGs assembly due to *E. coli* intervention and the malignant behavior of ICC were restored to some extent when anti‐IL6 was added. Additionally, our results showed that neither TPCA nor anti‐IL6 alone were sufficient to fully restore functional enhancement by *E. coli* stimulation. We selected ICA as a new drug with dual effects (inhibiting IL6 and NF‐κB) to significantly inhibit the production of SGs in ICC cells stimulated by *E. coli*. Our study is the first to provide a new perspective on the progression of ICC with hepatolithiasis and a new theoretical basis for the role of SGs in tumors.

## 4 Conclusion

*E. coli* enhances the malignancy of ICC by upregulating the expression of splicing factor eIF4A3, promoting the assembly of SGs, and leading to sustained activation of the NF‐κB pathway. cGLIS3 upregulation represents a promising diagnostic and therapeutic target for ICC and adds a new dimension to the functional importance of circRNA regulation in cancer. Our study reveals a novel mechanism and therapeutic strategy for the treatment of ICC.

## 5 Experimental Section

### Patients and Specimens

Intrahepatic cholangiocarcinoma and corresponding normal tissues were collected from 118 patients in the Department of Hepatobiliary Pancreatic Surgery, Fujian Provincial Hospital, between January 2016 and June 2020. Twelve pairs (tumor and normal tissues) were used for RNA‐seq analysis. Detailed clinicopathological data, such as sex, age, tumor size, and TNM stage, were obtained from hospital records. This study was approved by the Ethics Committee of Fujian Provincial Hospital (Fuzhou, China). Patients or their guardians signed an informed consent form prior to participation in the study.

### rRNA Sequencing

Bile samples derived from patients with hepatolithiasis‐related ICC (*n* = 20) were subjected to 16S rRNA sequencing at Gene Denovo Biotechnology Co.(Guangzhou, China);Microbial DNA was extracted using HiPure Soil DNA Kits (or HiPure Stool DNA Kits) (Magen, Guangzhou, China) according to the manufacturer's protocols. The 16S rDNA target region of the ribosomal RNA gene was amplified by PCR(95 for 5 min, followed by 30 cycles at 95 °C for 1 min, 60 °C for 1 min, and 72 °C for 1 min, and a final extension at 72 °C for 7 min). Fifty microliters mixture contained 10 µL of 5 × Q5@ Reaction Buffer, 10 µL of 5 × Q5@ High GC Enhancer, 1.5 µL of 2.5 mm dNTPs, 1.5 µL of each primer (10 µm), 0.2 µL of Q5@ High‐Fidelity DNA Polymerase, and 50 ng of template DNA. PCR reagents were purchased from New England Biolabs (USA). Amplicons were extracted from 2% agarose gels, purified using an AxyPrep DNA Gel Extraction Kit (Axygen Biosciences, Union City, CA, U.S.) according to the manufacturer's instructions, and quantified using an ABI StepOnePlus Real‐Time PCR System (Life Technologies, Foster City, USA). Purified amplicons were pooled in equimolar amounts and paired‐end sequenced (PE250) on an Illumina platform according to standard protocols.

### Cell Culture

ICC cell lines (HuCC‐T1, HCCC‐9810) were purchased from Cellcook Biotech (Guangzhou, China). HuCC‐T1 and HCCC‐9810 cells were cultured in RPMI‐1640 medium (Gibco). The media for all the cell lines contained 10% fetal bovine serum (Gibco, CA, USA), 100 U mL penicillin, and 100 µg mL streptomycin (Gibco, CA, USA), and cells were cultured in a humidified incubator with 5% CO_2_ at 37 °C. Based on previous studies, *E. coli* was used at an Multiplicity of infection (MOI) of 50:1.

### Bacterial Strain Culture

*E. coli* strain ATCC25922 (purchased from China General Microbiological Culture Collection Center) was sensitive to all the tested antimicrobial agents. *E. coli* was grown on Luria–Bertani (LB) medium and stored at 20 °C prior to use.

### RNA‐seq Analysis

Twelve pairs (tumor tissue and normal tissue) of HuccT1 cells (treated with PBS or *E.coli*) were used to perform circRNA‐seq analysis using SeqHealth Technology (Wuhan, China). Total RNAs were extracted from ICC cells using TRIzol reagent (Invitrogen, CA, USA). Total RNAs were used for stranded RNA sequencing library preparation using the KC‐Digital Stranded mRNA Library Prep Kit and the circRNA library for Illumina (Seqhealth Technology, Wuhan, China) according to the manufacturer's instructions. The kit eliminated duplication bias in the PCR and sequencing steps using a unique molecular identifier of eight random bases to label the pre‐amplified cDNA molecules. Library products corresponding to 200–500 bp were enriched, quantified, and sequenced on a DNBSEQ‐T7 sequencer (MGI Tech, Shenzhen, China) using the PE150 model.

### Screening for Differentially Expressed Genes and Enrichment Analysis

The “limma” R package was used to infilter differentially expressed genes (DEGs). Gene Ontology (GO) functional annotation and Kyoto Encyclopedia of Genes and Genomes (KEGG) pathway analysis of DEGs were conducted by using the “clusterProfiler” R package.

### RNA Extraction and Quantitative Real‐Time PCR (qRT‐PCR) Analysis

Total RNA from ICC tissues or cell lines was isolated using the TRIzol Reagent (Invitrogen, Carlsbad, CA, USA) according to the manufacturer's protocol. Reverse transcription was performed using a PrimeScript RT Reagent Kit (Takara, Dalian, China). Bulge‐loop miRNA RT‐qPCR Primers were used to determine miRNA levels. Real‐time PCR was performed using the StepOnePlus Real‐Time PCR System (Thermo Fisher Scientific). The program settings for the temperature cycling were as instructed by the manufacturer. Relative circRNA, mRNA, and miRNA expression levels were normalized to those of GAPDH or U6 using the 2^−DDCT^ method.

### Ribonuclease R (RNase R) Treatment

Total RNA was incubated for 30 min at 37 °C with or without 3 U µg^−1^ RNase R (Geenseed, Guangzhou, China) according to manufacturer's instructions and then the expression levels of cGLIS3 and GLIS3 mRNA were detected by qRT‐PCR.

### Actinomycin D Assay

The cells were treated with 2 µg mL^−1^ actinomycin D (Sigma–Aldrich) for different durations. Harvesting of cells and RNA extraction according to treatment time RNA expression levels were detected using qRT‐PCR.

### Immunohistochemistry (IHC)

The tumor tissues were fixed in 4% paraformaldehyde and embedded in paraffin. Tissue sections were deparaffinized, rehydrated, and subjected to antigen retrieval by heat treatment in a citrate buffer. Samples were blocked with 5% BSA for 1 h. Primary antibodies were incubated overnight at 4 °C, followed by incubation with secondary antibodies at room temperature for 1 h. A DAB solution was used for the chromogenic reactions.

### Immunofluorescence (IF)

Samples were fixed in 4% paraformaldehyde at room temperature for 15 min, then permeabilized with 0.2% Triton X‐100 for 10 min, and blocked with 5% BSA at room temperature for 1 h. Primary antibodies were incubated overnight at 4 °C, followed by incubation with fluorescent secondary antibodies at room temperature for 1 h. Samples were mounted after staining with DAPI. The antibodies were diluted in 1% bovine serum albumin (BSA) and 0.3% Triton‐X 100 in 1× PBS. For immunofluorescence, cells were incubated with rabbit anti‐hnRNPA1(Cell Signaling Technology, USA) and mouse anti‐G3BP1(Cell Signaling Technology, USA) primary antibodies overnight at 4 °C or 2 h at room temperature, followed by three washes with 1 × PBS. The cells were then incubated with Alexa Fluor 594 goat antirabbit (Cell Signaling Technology, Danvers, MA, USA) and Alexa Fluor 555 goat antimouse (Cell Signaling Technology) secondary antibodies for 2 h at room temperature, followed by three washes with 1 × PBS. Nuclei were stained with DAPI. Quantification of stress granule formation to quantify SG formation in cells, 60× images of at least three to five random fields of view on the coverslip were used for analysis from at least three independent experiments. Co‐localization of G3BP1‐positive or hnRNPA1‐positive puncta in the cytoplasmic fractions was counted as SG‐positive cells by two observers blinded to the conditions using ImageJ software and then averaged.

### Fluorescent In Situ Hybridization (FISH)

Specific fluorescent‐labeled cGLIS3, G3BP1, and hnRNPA1 probes were designed and synthesized by Service Bio (Wuhan, China). After fixation, permeabilization, and prehybridization, samples were hybridized in a hybridization buffer with the probes at 37 °C overnight. The hybridization buffer was then gradually washed off with 4× SSC (including 0.1% Tween‐20), 2× SSC, and 1× SSC at 42 °C. Nuclei were counterstained with DAPI. Images were captured using a confocal microscope in Servicebio (Wuhan, China).

### Western Blot Analysis

Briefly, proteins were isolated from ICC cells and tumor tissues using RIPA buffer (Solarbio, Beijing, China) supplemented with proteinase and phosphatase inhibitors. Protein concentration was determined using the BCA reagent (Beyotime, Beijing, China). Proteins were separated on sodium dodecyl sulfate‐polyacrylamide gels and transferred onto polyvinylidene difluoride (PVDF) membranes (Merck Millipore). After the membranes were blocked in 5% skim powdered milk for 1 h, they were incubated with primary antibodies overnight at 4 °C. The membranes were then incubated with secondary antibodies at room temperature for 1 h. Targeted proteins were detected using the Pierce ECL Western Blotting Kit (Thermo Fisher Scientific, MA, US) with a ChemiDoc MP Imaging System (Bio‐Rad, CA, USA).

### Agarose Gel Electrophoresis

Nucleic acid samples were loaded onto 2% (w/v) agarose gels and separated by electrophoresis in TAE running buffer at 120 V for 30 min. Gel images were visualized using a ChemiDoc MP Imaging System (Bio‐Rad, CA, USA).

### Microtumor Spheroid‐Based Migration Assays

ICC cell lines incubated with PBS or *E. coli* for 4 h in indirect co‐culture models were used to perform 3D tumor spheroid formation (2000 cells per well) in a 96‐well ultra‐low attachment round‐bottom plate (Corning, NY, USA). After tumor spheroid formation (24 h), the tumor spheroids were transferred to flat‐bottomed 96‐well plates (Corning, NY, USA) coated with 0.1% (v/v) gelatin (Sigma–Aldrich, MO, USA) for further migration assays. Images were obtained and the migration area was recorded using a microscope and quantified using ImageJ software.

### 5.1 Migration and Invasion Assay

ICC cell lines were incubated with PBS or *E. coli* for 4 h in advance using an indirect co‐culture model. For migration assay, the 2.5 × 10^5^ cells suspended in 100 µL medium with 1% serum were seeded in the upper chamber of transwell chambers (Corning, USA), and 800 µL fresh medium with 10% serum were added to the lower chamber. For invasion assay, 100 µL diluted Matrigel (Matrigel: PBS = 1:9) was added in the upper chamber of transwell chambers (Corning, USA) for 2 h. Before seeding the cells, the Matrigel was discarded. After incubation for 24 h at 37 °C, the cells in the upper chamber were fixed with 4% paraformaldehyde, followed by staining with 0.1% crystal violet. The number of migrated cells was quantified by counting in five fields (100× magnification).

### Indirect Co‐Culture Assay

*E. coli* strains were grown in LB for 6 h to an OD600 of ≈1.5–2. Biofil tissue culture plate insert chambers six‐wells plates (0.1 µm pore size) were used for the indirect co‐culture assay. ICC was seeded in the lower layer, whereas *E. coli* was seeded in the upper layer. Before co‐incubating *E. coli* with ICC cells, ICCs (1 × 10^6^ ICC cells were seeded in a 6‐well plate. In all experiments, ICC cells infected with *E. coli* at low multiplicities of infection (MOI) were used; a bacterial cell suspension was added to serum‐free DMEM, after which the medium was replaced with DMEM‐containing *E*. *coli* strains. The cells were infected with *E. coli* (MOI = 1:50) for 4 h.

Subsequently, the *E. coli* in the upper layer was removed, and the ICC cells were replaced with fresh culture medium and used to perform further experiments. For colony formation assays, after gemcitabine treatment, as indicated, ICC was fixed with 4% paraformaldehyde for 15–20 min and stained with crystal violet for 15 min. Finally, images of the cells were recorded under a fluorescence microscope, as previously reported.^[^ ^41^ ^]^

### RNA Immunoprecipitation (RIP) Assays

The RIP assay was performed using the Magna RIP RNA‐binding protein immunoprecipitation kit (Merck Millipore, MA, USA), following the manufacturer's protocol. One milliliter of RIP lysis buffer containing protease and RNase inhibitors was used to lyse the cells (≈1 × 107). The lysates were incubated with IgG (Abclonal Rabbit Control IgG AC005), anti‐G3BP1, and anti‐hnRNPA1 antibody‐coated beads (Millipore) at 4 °C overnight. Next, the RNA‐protein complexes were isolated by incubating cell lysates with the protein A/G magnetic beads at 4 °C for 1 h. After proteinase K digestion, protein‐bound RNAs were extracted by phenol/chloroform/isoamyl alcohol (125:24:1) (Solarbio) and reverse transcribed to cDNA. The RNA levels of target genes were then evaluated using qRT‐PCR. Co‐precipitated RNA was detected by qRT‐PCR.

### RNA Antisense Pull‐Down Assay

For RNA pull‐down assay, a BersinBio RNA Antisense Purification (RAP) kit (catalog no. Bes5103, BersinBio, China) was used following the manufacturer's protocol. Biotin‐labeled circGLIS3 probe was synthesized by BersinBio (Guangzhou, China). Briefly, cross‐linked cells were lysed, sonicated, and then hybridized with the probe for 4 h at 37 °C. The hybridization mixture was then treated with magnetic beads for 1 h. After that, the bound proteins were eluted and collected, then prepared for silver staining and mass spectrometry analysis.

### Organoids Culture

Organoids were derived from tumor tissues of patients with ICC. Tumor tissues were cut into small pieces, digested using MasterAim tissue enzymatic solution I (StemCell Technologies, Vancouver, Canada) and advanced DMEM/F12 (Gibco, CA, USA) for 1 h, and further digested in MasterAim tissue enzymatic solution II (StemCell Technologies, Vancouver, Canada). The digestion was stopped by adding advanced DMEM/F12 containing 10% fetal bovine serum (FBS). Cells were then resuspended in Matrigel (Corning, NY, USA) and plated in 24‐well plates at 37 °C for 30 min to allow solidification. ICC organoid complete medium (BioGenous, Hangzhou, China) was supplemented for organoid growth. Organoid formation was assisted by Keystone Co. Ltd. (Keystone, Fuzhou, China).

### RNC Sequencing

ICC cells that were indirectly co‐cultured with *E. coli* for 4 h and control ICC cells treated with PBS for 4 h were used to perform RNC sequencing using Gene Denovo Biotechnology Co. (Guangzhou, China). To immobilize initiating ribosomes, harringtonine (Abcam, Cambridge, MA, USA) was added to cell culture medium to a final concentration of 2 µg mL^−1^. The cells were then incubated for 120 s with harringtonin. Next, to block translational elongation, cycloheximide (Sigma, St Louis, MO, USA) was added to cell culture medium to a final concentration of 100 µg mL^−1^. The cells were mixed thoroughly and immediately lysed. The resuspended extracts were immediately loaded onto a 1 m sucrose cushion prepared in a polysome buffer containing 0.1 U mL^−1^ SuperaseIn. Ribosomes were pelleted by centrifugation for 4 h at 70 000 rpm, 4 °C in a TLA‐110 rotor. The liquid was removed, and the pellet was resuspended in 570 mL 10 mm Tris (pH 7), followed by the immediate addition of 30 mL 20% SDS. The sample was heated to 65 °C and RNA was extracted using two rounds of acid phenol/chloroform followed by chloroform alone. RNA was precipitated from the aqueous phase by the addition of sodium acetate to a final concentration of 300 mm, followed by the addition of at least one volume of isopropanol. Precipitation was carried out at −30 °C for 30 min and RNA was then pelleted by centrifugation for 30 min at 20 000 3 g, 4 °C. The supernatant was discarded, the pellet was air‐dried, and the RNA was resuspended in 150 mL Tris (pH 7). The typical RNA yield was 100–200 mg. After total RNA extraction, rRNA was removed using a previously reported method. rRNAs were removed to retain the mRNAs and ncRNAs. The enriched mRNAs and ncRNAs were fragmented into short fragments using a fragmentation buffer and reverse‐transcribed into cDNA with random primers. Second‐strand cDNA was synthesized using DNA polymerase I, RNase H, dNTP (dUTP instead of dTTP), and buffer. Next, the cDNA fragments were purified using a QiaQuick PCR extraction kit (Qiagen, Venlo, Netherlands), end‐repaired, poly(A) added, and ligated to Illumina sequencing adapters. Then UNG (Uracil‐N‐Glycosylase) was used to digest the second‐strand cDNA. The digested products were size‐selected by agarose gel electrophoresis, PCR‐amplified, and sequenced using an Illumina HiSeqTM 4000 (or other platforms) by Gene Denovo Biotechnology Co. (Guangzhou, China).

### Animal Experiments

For animal experiments, four 6 weeks old male BALB/c nude mice were housed in laminar flow cabinets under specific pathogen‐free conditions, with food and water provided ad libitum. The mice were randomly divided into groups according to the different treatments. For the subcutaneous model, ICC cells indirectly co‐cultured with *E. coli* for 4 h were injected subcutaneously into the right axilla (1 × 10^7^ cells per mouse). Six days after the injection, *E. coli* or PBS was administered via multipoint intratumoral injection twice per week for 4 weeks. Chemotherapeutic agents and ICA were administered via intraperitoneal injection twice per week for 2 weeks. The tumors were surgically obtained after 2 weeks of chemotherapeutic agents treatments, and gross images and hematoxylin and eosin (H&E) staining of the tissue sections were used to evaluate tumor multiplicity. The following formula was used to determine tumor volume: 0.5 × (length × width^2^). For the lung metastasis model, ICC cells were indirectly co‐cultured with PBS or *E. coli* (MOI of 50:1) and injected into the tail vein of BALB/c nude mice (2 × 10^6^ cells per mouse). Chemotherapeutic agents (gemcitabine and cisplatin) and ICA were administered by intraperitoneal injection twice per week for 3 weeks. Subsequently, bioluminescence imaging was used to evaluate tumor metastasis^[^ ^42^ ^]^ before sacrificing.

All animal experiments were conducted in accordance with the Laboratory Animal Care guidelines (http://www.aaalac.org) and approved by the Institutional Animal Care and Use Committee of Fujian Provincial Hospital. Four to six weeks old male BALB/c nude mice were purchased from SLAC Laboratory Animal Co., Ltd. (Shanghai, China). To evaluate the therapeutic efficacy of GP in combination with ICA, all mice except the control group were treated with ICA (35 mg kg^−1^), gemcitabine (40 mg kg^−1^), or cisplatin (1 mg kg^−1^) via intraperitoneal injection twice weekly for 3 weeks. ICA was donated by Hangzhou Zhongmei Huadong Pharma Ceutical Co., Ltd.

### Statistical Analysis

GraphPad Prism 9.0 and SPSS 23.0 were applied for statistical analysis. Data between two groups were assessed using Student's *t‐*test or Mann–Whitney U test as appropriate. Data in multiple groups were evaluated using analysis of variance (ANOVA). Overall survival (OS) and progression‐free survival (PFS) were assessed with the Kaplan−Meier method and compared by the Log‐rank test. The χ^2^ test was used to evaluate the association between cGLIS3 levels and clinicopathological parameters of patients. Representative data are shown as the mean ± standard deviation (SD). A *p*‐value <0.05 was considered to be statistically significant. *p < 0.05, **p < 0.01, ***p < 0.001, and *p* ≥ 0.05 mean no significance, ns.

### Ethics Approval Statement

All clinical tissue samples were collected from the Department of Hepatobiliary Surgery at Fujian Provincial Hospital. This study was approved and authorized by the Ethics Committee of Fujian Provincial Hospital (K2021‐08‐018). All animal study procedures and experiments were approved by the Institutional Animal Care and Use Committee (IACUC) of Fujian Medical University (IACUC FJMU 2023‐0177) and performed in accordance with the Guidelines for the Care and Use of Laboratory Animals.

## Conflict of Interest

The authors declare no conflict of interest.

## Author Contributions

Z.‐W.C., F.‐P.K., and C.‐Y.L. contributed equally to this work. S.C. and Z.‐W.W. designed and directed the study. F.‐P.K. and Z.‐W.C. performed experiments and wrote the manuscript. C.‐Y.L. collected the tissues. C.‐K.X., G.L., and H.‐Y.L. performed data analysis. Y.‐D.W. assisted with experiments. J.‐F.H. provided technical assistance. All the authors have read and approved the final version of the manuscript.

## Supporting information

Supporting Information

## Data Availability

The data that support the findings of this study are available from the corresponding author upon reasonable request.
