# Supplementary material for: Escherichia coli‐Induced cGLIS3‐Mediated Stress Granules Activate the NF‐κB Pathway to Promote Intrahepatic Cholangiocarcinoma Progression
Source: Adv Sci (Weinh). 2024 Feb 17;11(16):2306174. doi: 10.1002/advs.202306174 (PMC11040339; doi:10.1002/advs.202306174)
Supplement: Supplementary file 1 — Supporting Information [file ADVS-11-2306174-s001.pdf]

ADVANCED SCIENCE
Open Access

Supporting Information

for *Adv. Sci.*, DOI 10.1002/advs.202306174

Escherichia coli-Induced cGLIS3-Mediated Stress Granules Activate the NF-κB Pathway to Promote Intrahepatic Cholangiocarcinoma Progression

Feng-Ping Kang, Zhi-Wen Chen, Cheng-Yu Liao, Yong-Ding Wu, Ge Li, Cheng-Ke Xie, Hong-Yi Lin, Long Huang, Yi-Feng Tian, Zu-Wei Wang and Shi Chen**

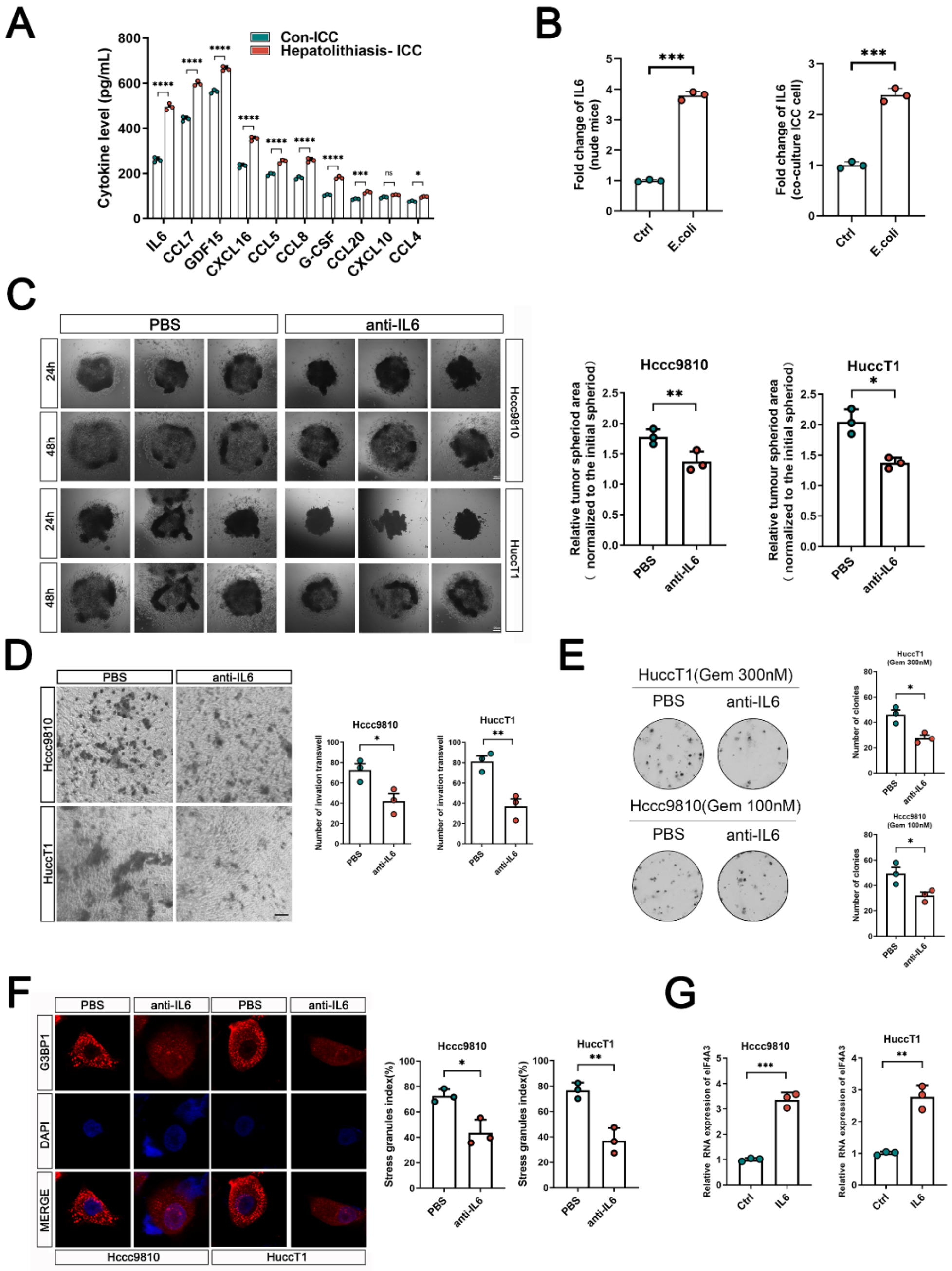

A
Con-ICC
Hepatolithiasis- ICC
Cytokine level (pg/mL)
IL6
CCL7
GDF15
CXCL16
CCL5
CCL8
G-CSF
CCL20
CXCL10
CCL4
B
Fold change of IL6 (nude mice)
Fold change of IL6 (co-culture ICC cell)
Ctrl
E.coli
C
PBS
anti-IL6
24h
48h
Hccc9810
HuccT1
Relative tumor spheriod area (normalized to the initial spheriod)
D
Number of invation transwell
E
HuccT1(Gem 300nM)
Hccc9810(Gem 100nM)
Number of clonies
F
G3BP1
DAPI
MERGE
Stress granules index(%)
G
Relative RNA expression of eIF4A3
Ctrl
IL6

Figure S1

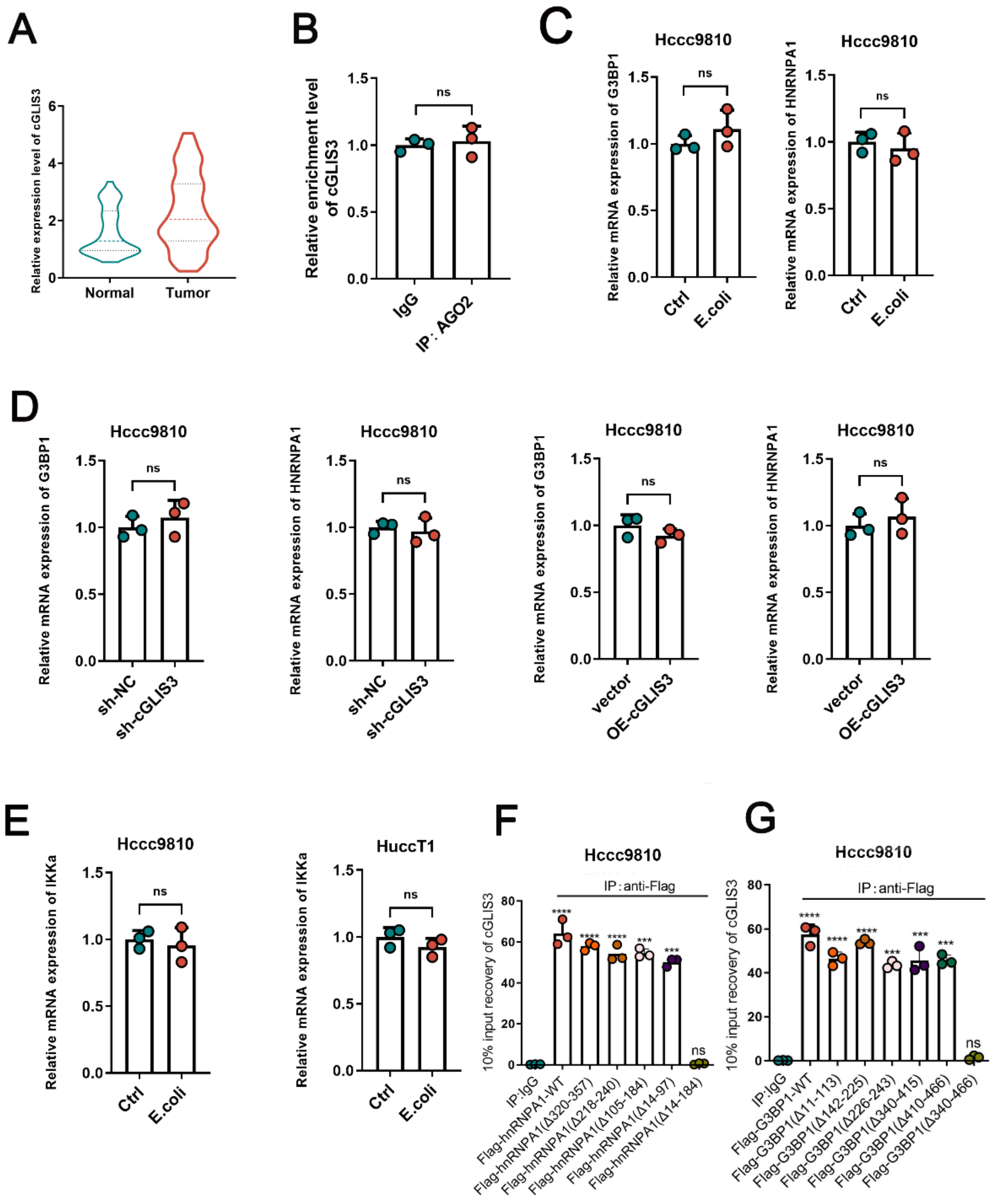
A
Relative expression level of cGLIS3
Normal
Tumor
B
Relative enrichment level of cGLIS3
ns
IgG
IP: AGO2
C
Hccc9810
Relative mRNA expression of G3BP1
Relative mRNA expression of HNRNPA1
Ctrl
E.coli
D
sh-NC
sh-cGLIS3
vector
OE-cGLIS3
E
Relative mRNA expression of IKKa
HuccT1
F
10% input recovery of cGLIS3
IP: anti-Flag
IP:IgG
Flag-hnRNPA1-WT
Flag-hnRNPA1(Δ320-357)
Flag-hnRNPA1(Δ218-240)
Flag-hnRNPA1(Δ105-184)
Flag-hnRNPA1(Δ14-97)
Flag-hnRNPA1(Δ14-184)
G
Flag-G3BP1-WT
Flag-G3BP1(Δ11-113)
Flag-G3BP1(Δ142-225)
Flag-G3BP1(Δ226-243)
Flag-G3BP1(Δ340-415)
Flag-G3BP1(Δ410-466)
Flag-G3BP1(Δ340-466)

Figure S2

Figure S1 The anti-IL6 partially inhibits the progression-promoting effects of E.coli.

A. Changes in cytokines in the bile of ICC patients with hepatolithiasis or without hepatolithiasis.

B. ELISA quantification of IL-6 level of the tumors homogenate from the subcutaneous model injecting by Huccc9810 cells that pretreated with E. coli or PBS (left); ELISA quantification of IL-6 level in the co-culture medium from the indirect co-culture model between Huccc9810 cells and E.coli (Right);

C.3D tumor migration assay of Hccc9810 and HuccT1 cells cultured with PBS or anti-IL6, after treatment with E. coli. Quantified in the right panel. Scale bar: 100 μm.

D. Transwell assay of Hccc9810 and HuccT1 cells cultured with PBS or anti-IL6, after pretreated with E. coli. quantified in the right panel.

E. Colony formation assay of Hccc9810 and HuccT1 cells cultured with PBS or anti-IL6, after pretreated with E. coli and the numbers of colonies are summarized on the right.

F. The percentage of cells with stress granules (G3BP1+) of Hccc9810 and HuccT1 cells cultured with PBS or anti-IL6, after pretreated with E. coli.

G. qRT-PCR detection of relative mRNA expression of eIF4A3 in Hccc9810 and HuccT1 cells treated with IL-6 or PBS.

Figure S2 Relationship between cGLIS3 and hnRNANPA1/G3BP1

A. Expression levels of cGLIS3 were examined between ICC tumor tissue and normal tissue.

B. The interaction of cGLIS3 and AGO2 based on RIP experiment.

C. The coding potential of cGLIS3 was predicted based on the circRNAD database.

D. Effect of overexpression or knockdown of cGLIS3 on hnRNPA1/G3BP1 mRNA level, as shown by the qPCR experiment.

E. Effect of pretreated with E. coli or PBS on IKKA mRNA level of the Hccc9810 and HuccT1 cells, as shown by the qPCR experiment.

F. Binding of the Full-length or different hnRNPA1 truncations and cGLIS3, RIP experiment was performed using anti-flag.

G. Binding of the Full-length or different G3BP1 truncations and cGLIS3, RIP experiment was performed using anti-flag.

Table S1 . Baseline characteristics of patients with ICC according to cGLIS3 expression levels**.**

Characteristic	cGLIS3 low-expression n=59	cGLIS3 high-expression n=59	*p* value
Sex			0.854
male	31(52.5)	32(54.2)	
female	28(47.5)	27(45.8)	
Age			0.853
<60	26(44.1)	27(45.8)	
≥60	33(55.9)	32(54.2)	
BMI			0.847
<25	39(66.1)	38(64.4)	
⩾25	20(33.9)	21(35.6)	
Hepatolithisasis			<0.001
no	48(81.4)	17(28.8)	
yes	11(18.6)	42(71.2)	
Bile culture positive	9(15.3)	36(61.0)	<0.001
E.*Coli* positive	8(13.6)	33(55.9)	<0.001
CA19-9, UI/mL	93.5(53.9-139.1)	151.7(83.1-283.9)	<0.001
Differentiation			<0.001
well	8(13.6)	4(6.8)	
moderate	39(66.1)	19(32.2)	
poor	12(20.3)	36(61.0)	
vascular invasion	25(42.4)	36(61.0)	0.043
Tumor number			0.018
single	55(93.2)	46(78.0)	
multiple	4(6.8)	13(22.0)	
Tumor size, cm	4.5(3.6-5.7)	5.8(4.6-6.7)	<0.001
Lymph node metastasis	32(54.2)	49(83.1)	0.001
Distant metastasis	3(5.1)	13(22.0)	0.007
AJCC			0.003
I	12(20.3)	3(5.1)	
II	13(22.0)	7(11.9)	
III	31(52.5)	36(61.0)	
IV	3(5.1)	13(22.0)	

Table S2. Univariate and multivariate analysis for PFS.

Variables	Univariate		Multivariate	
	OR(95%CI)	*p* value	OR(95%CI)	*p* value
Sex				
male	REF	0.481		
female	1.154(0.775-1.717)			
Age		0.267		
<60	REF			
≥60	1.256(0.839-1.880)			
BMI		0.434		
<25	REF			
⩾25	1.178(0.781-1.776)			
Hepatolithisasis		0.001		0.631
no	REF			
yes	2.042(1.356-3.075)			
Bile culture positive	2.562(1.669-3.932)	<0.001		
Escherichia coli	2.683(1.735-4.149)	<0.001	1.875(1.187-2.961)	0.007
Klebsiella pneumoniae	1.506(0.833-2.723)	0.175		
Enterococus	1.324(0.611-2.870)	0.476		
Staphy	1.324(0.611-2.870)	0.396		
CA19-9, UI/mL		0.005		0.526
<100	REF			
≥100	1.791(1.191-2.693)			
Differentiation		0.048		0.837
well	REF			
moderate	1.623(0.816-3.227)			
poor	2.283(1.130-4.609)			
vascular invasion		0.066		
no	REF			
yes	1.453(0.975-2.165)			
AJCC		0.048		0.007
I	REF			
II	1.586(0.726-3.466）		1.526(0.697-3.341)	0.29
III	3.259（1.657-6.412）		2.888(1.452-5.745)	0.003
IV	46.433(18.061-119.377)		35.177(13.405-92.310)	<0.001

Table S3. Univariate and multivariate analysis for OS.

Variables	Univariate		Multivariate	
	OR(95%CI)	*p* value	OR(95%CI)	*p* value
Sex				

female	REF	0.701		
male	1.088(0.708-1.671)			
Age		0.36		
<60	REF			
≥60	1.226(0.792-1.898)			
BMI		0.458		
<25	REF			
⩾25	1.183(0.759-1.845)			
cGLIS3 expression		0.008		0.449
low-expression	REF			
high-expression	1.745(1.170-2.603)			
Hepatolithisasis		0.002		0.108
no	REF			
yes	2.000(1.286-3.110)			
Bile culture assay	2.489(1.586-3.905)	<0.001		
E.Coli positive	**2.468(1.567-3.885)**	**<0.001**		**0.069**
K. pneumoniae positive	1.227(0.649-2.321)	0.529		
Enterococus positive	1.021(0.443-2.352)	0.961		
Staphy positive	1.777(0.648-4.871)	0.264		
CA19-9, UI/mL		0.001		0.104
<100	REF			
≥100	2.083(1.329-3.262)			
Differentiation		0.151		
well	REF			
moderate	1.382(0.647-2.953)	0.404		
poor	1.948(0.897-4.232)	0.092		
vascular invasion		0.074		
no	REF			
yes	1.485(0.962-2.291)			
AJCC		<0.001		
I	REF		REF	
II	1.622(0.636-4.137）	0.311	1.622(0.636-4.137）	0.311
III	3.598(1.608-8.050)	0.002	3.598(1.608-8.050)	0.002
IV	60.210(20.349-178.157)	<0.001	60.210(20.349-178.157)	<0.001

Table S4. The sequences of all plasmids

Name	Sequence (5'- 3')

cGLIS3-<br>WT	GCTTATAACCCACACCATGGATAACTTATTGGACTTTGCCTGAAAGGAGT<br>TGACATTGGATATTTGACCGTCTTGGCCACAGGTTTTTCAGAATGAATG[illegible]<br>ATGCAGCATGAGTCTCCACCGGACATCGGGAACCCCACAGGGGCCTAG[illegible]<br>AGTGGTCATCACATTCCTGCCATCCGAGCCCACTCCGGGACTCCTGGC[illegible]<br>CTGTGGCAGCACATCGAGTCCCACTATGGCAAGCCTTGCTAACAACCT[illegible]<br>AGATGCCCTCAGGAGGAGGGATGGCTCCTCAGAACAACGTGGCTGAGA<br>TCCATCTGCCTGCCTTAAGCCCCAGGAGACAAATGCTCACCAATGGGAA<br>ATTCCAGGTCACCCAGGCTGGAGGCATGTCAGGGTCACATACTTTAAA[illegible]<br>CAGCAGGAGTTTGGAAGCCCTTTTCCTCCAAATCCTGGGAAAG
cGLIS3-<br>HR1-<br>mut	GCTTATAACCCACACCATGGATAACTTATTGGACTTTGCCTGAAAGGA<br>GTCATTAGTGACATTGGATATTTGACCGTCTTGGCCACAGGTTTTTCA<br>GAATGAATGGAAGATCATGCAGCATGAGTCTCCACCGGACATCGGGA<br>ACCCCACAGGGGCCTAGGATGGTCAGTGGTCATCACATTCCTGCCATC<br>CGAGCCCACTCCGGGACTCCTGGCCCCTCGCCCTGTGGCAGCACATC<br>GAGTCCCACTATGGCAAGCCTTGCTAACAACCTCCATCTCAAGATGCC<br>CTCAGGAGGAGGGATGGCTCCTCAGAACAACGTGGCTGAGAGCCGC<br>ATCCATCTGCCTGCCTTAAGCCCCAGGAGACAAATGCTCACCAATGGG<br>AAGCCGCGATTCCAGGTCACCCAGGCTGGAGGCATGTCAGGGTCACA<br>TACTTGCCCTAACCCTACTACTGAGTTTGGAAGCCCTTTTCCTCCAAA<br>TCCTGGGAAAG
cGLIS3-<br>HR2-<br>mut	GCTTATAACCCACACCATGGATAACTTATTGGACTTTGCCTGAAAGGA<br>GTCATTAGTGACATTGGATATTTGACCGTCTTGGCCACAGGTTTTTCA<br>GAATGAATGGAAGATCATGCAGCATGAGTCTCCACCGGACATCGGGA<br>ACCCCACAGGGGCCTAGGATGGTCAGTGGTCATCACATTCCTGCCATC<br>CGAGCCCACTCCGGGACTCCTGGCCCCTCGCCCTGTGGCAGCACATC<br>GAGTCCCACTATGGCAAGCCTTGCTAACAACCTCCATCTCAAGATGCC<br>CTCAGGAGGAGGGATGGCTCCTCAGAACAACGTGGCTGAGAGCCTA<br>CGAACGAGTAAGTAAGGCCTAAAACTTCTCACCCGTAGACAACCGTT<br>TCCTAATATCGGAACTTGACCCCAGGCTGGAGGCATGTCAGGGTCAC<br>ATACTTTAAAGCCAAAGCAGCAGGAGTTTGGAAGCCCTTTTCCTCCA<br>AATCCTGGGAAAG
cGLIS3-<br>HR3-<br>mut	TAGGCGCCAAACACAACGTTCGCCAGGCGGTTCAGGGTAATGAAAG<br>GAGTCATTAGTGACATTGGATATTTGACCGTCTTGGCCACAGGTTTTT<br>CAGAATGAATGGAAGATCATGCAGCATGAGTCTCCACCGGACATCGG<br>GAACCCCACAGGGGCCTAGGATGGTCAGTGGTCATCACATTCCTGCC<br>ATCCGAGCCCACTCCGGGACTCCTGGCCCCTCGCCCTGTGGCAGCAC<br>ATCGAGTCCCACTATGGCAAGCCTTGCTAACAACCTCCATCTCAAGAT<br>GCCCTCAGGAGGAGGGATGGCTCCTCAGAACAACGTGGCTGAGAGC<br>CGCATCCATCTGCCTGCCTTAAGCCCCAGGAGACAAATGCTCACCAAT<br>GGGAAGCCGCGATTCCAGGTCACCCAGGCTGGAGGCATGTCAGGGTC<br>ACATACTTTAAAGCCAAAGCAGCAGGAGTTTGGAAGCCCTTTTCCTC<br>CAAATCCTGGTCCCT

cGLIS3-HR4-mut	GCTTATAACCCACACCATGGATAACTTATTGGACTTTGCCTGAAAGGA GTCATTAGTGACATTGGATATTTGACCGTCTTGGCCACAGGTTTTTCA GAATGAATGGAAGATCATGCAGCATGAGTCTCCACCGGACATCGGGA ACCCCACAGGGGCCTAGGATGGTCAGTGGTCATCACATTCCTGCCATC CGAGCCCACTCCGGGACTCCTGGCCCCTCGCCCTGTGGCAGCACATC GAGTCCCACTATGGCAAGCCTTGCTAACAACCTCCATCTCAATCGTAA ATCAGGAGGAGGGATGGCTCCTCAGAACAACGTGGCTGAGAGCCGC ATCCATCTGCCTGCCTTAAGCCCCAGGAGACAAATGCTCACCAATGGG AAGCCGCGATTCCAGGTCACCCAGGCTGGAGGCATGTCAGGGTCACA TACTTTAAAGCCAAAGCAGCAGGAGTTTGGAAGCCCTTTTCCTCCAA ATCCTGGGAAAG
cGLIS3-HR5-mut	GCTTATAACCCACACCATGGATAACTTATTGGACTTTGCCTGAAAGGA GTCATTAGTGACATTGGATATTTGACCGTCTTGGCCACAGGTTTTTCA GAATGAATGGAAGATCATGCAGCATGAGTCTCCACCGGACATCGGGA ACCCCACAGGGGCCTAGGATGGTCATGTTGACGACACGGAATGCCAT CCGAGCCCACTCCGGGACTCCTGGCCCCTCGCCCTGTGGCAGCACAT CGAGTCCCACTATGGCAAGCCTTGCTAACAACCTCCATCTCAAGATGC CCTCAGGAGGAGGGATGGCTCCTCAGAACAACGTGGCTGAGAGCCG CATCCATCTGCCTGCCTTAAGCCCCAGGAGACAAATGCTCACCAATGG GAAGCCGCGATTCCAGGTCACCCAGGCTGGAGGCATGTCAGGGTCAC ATACTTTAAAGCCAAAGCAGCAGGAGTTTGGAAGCCCTTTTCCTCCA AATCCTGGGAAAG
hnRNPA1(del320-357aa)	ATGTCTAAGTCAGAGTCTCCTAAAGAGCCCGAACAGCTGAGGAAGCT CTTCATTGGAGGGTTGAGCTTTGAAACAACTGATGAGAGCCTGAGGA GCCATTTTGAGCAATGGGGAACGCTCACGGACTGTGTGGTAATGAGA GATCCAAACACCAAGCGCTCCAGGGGCTTTGGGTTTGTCACATATGCC ACTGTGGAGGAGGTGGATGCAGCTATGAATGCAAGGCCACACAAGGT GGATGGAAGAGTTGTGGAACCAAAGAGAGCTGTCTCCAGAGAAGAT TCTCAAAGACCAGGTGCCCACTTAACTGTGAAAAAGATATTTGTTGGT GGCATTAAAGAAGACACTGAAGAACATCACCTAAGAGATTATTTTGA ACAGTATGGAAAAATTGAAGTGATTGAAATCATGACTGACCGAGGCA GTGGCAAGAAAAGGGGCTTTGCCTTTGTAACCTTTGACGACCATGAC TCCGTGGATAAGATTGTCATTCAGAAATACCATACTGTGAATGGCCAC AACTGTGAAGTTAGAAAAGCCCTGTCAAAGCAAGAGATGGCTAGTGC TTCATCCAGCCAAAGAGGTCGAAGTGGTTCTGGAAACTTTGGTGGTG GTCGTGGAGGTGGTTTCGGTGGGAATGACAACTTCGGTCGTGGAGGA AACTTCAGTGGTCGTGGTGGCTTTGGTGGCAGCCGTGGTGGTGGTGG ATATGGTGGCAGTGGGGATGGCTATAATGGATTTGGTAATGATGGTGG TTATGGAGGAGGCGGCCCTGGTTACTCTGGAGGAAGCAGAGGCTATG GAAGTGGTGGACAGGGTTATGGAAACCAGGGCAGTGGCTATGGCGG GAGTGGCAGCTATGACAGCTATAACAACGGAGGCGGAGGCGGCTTTG GCGGTGGTAGTGGAAGCAATTTTGGAGGTGGTGGAAGCTACAATGAT TTTGGGAATTACAACGGCGGTTCCAGCAGCAGCAGTAGCTATGGCAG TGGCAGAAGATTTTAA

hnRNP A1(del218-240aa)	ATGTCTAAGTCAGAGTCTCCTAAAGAGCCCGAACAGCTGAGGAAGCTCTTCATTGGAGGGTTGAGCTTTGAAACAACTGATGAGAGCCTGAGGAGCCATTTTGAGCAATGGGGAACGCTCACGGACTGTGTGGTAATGAGAGATCCAAACACCAAGCGCTCCAGGGGCTTTGGGTTTGTCACATATGCCACTGTGGAGGAGGTGGATGCAGCTATGAATGCAAGGCCACACAAGGTGGATGGAAGAGTTGTGGAACCAAAGAGAGCTGTCTCCAGAGAAGATTCTCAAAGACCAGGTGCCCACTTAACTGTGAAAAAGATATTTGTTGGTGGCATTAAAGAAGACACTGAAGAACATCACCTAAGAGATTATTTTGAACAGTATGGAAAAATTGAAGTGATTGAAATCATGACTGACCGAGGCAGTGGCAAGAAAAGGGGCTTTGCCTTTGTAACCTTTGACGACCATGACTCCGTGGATAAGATTGTCATTCAGAAATACCATACTGTGAATGGCCACAACTGTGAAGTTAGAAAAGCCCTGTCAAAGCAAGAGATGGCTAGTGCTTCATCCAGCCAAAGAGGTCGAAGTGGTTCTGGAAACTTTGGTGGTGGTCGTGGAGGTGGTTTCGGTGGGAATGACAACTTCGGTGGGGATGGCTATAATGGATTTGGTAATGATGGTGGTTATGGAGGAGGCGGCCCTGGTTACTCTGGAGGAAGCAGAGGCTATGGAAGTGGTGGACAGGGTTATGGAAACCAGGGCAGTGGCTATGGCGGGAGTGGCAGCTATGACAGCTATAACAACGGAGGCGGAGGCGGCTTTGGCGGTGGTAGTGGAAGCAATTTTGGAGGTGGTGGAAGCTACAATGATTTTGGGAATTACAACAATCAGTCTTCAAATTTTGGACCCATGAAGGGAGGAAATTTTGGAGGCAGAAGCTCTGGCCCCTATGGCGGTGGAGGCCAATACTTTGCAAAACCACGAAACCAAGGTGGCTATGGCGGTTCCAGCAGCAGCAGTAGCTATGGCAGTGGCAGAAGATTTTAA
hnRNP A1(del105-184aa)	ATGTCTAAGTCAGAGTCTCCTAAAGAGCCCGAACAGCTGAGGAAGCTCTTCATTGGAGGGTTGAGCTTTGAAACAACTGATGAGAGCCTGAGGAGCCATTTTGAGCAATGGGGAACGCTCACGGACTGTGTGGTAATGAGAGATCCAAACACCAAGCGCTCCAGGGGCTTTGGGTTTGTCACATATGCCACTGTGGAGGAGGTGGATGCAGCTATGAATGCAAGGCCACACAAGGTGGATGGAAGAGTTGTGGAACCAAAGAGAGCTGTCTCCAGAGAAGATTCTCAAAGACCAGGTGCCCACTTAACTGTGGAGATGGCTAGTGCTTCATCCAGCCAAAGAGGTCGAAGTGGTTCTGGAAACTTTGGTGGTGGTCGTGGAGGTGGTTTCGGTGGGAATGACAACTTCGGTCGTGGAGGAAACTTCAGTGGTCGTGGTGGCTTTGGTGGCAGCCGTGGTGGTGGTGGATATGGTGGCAGTGGGGATGGCTATAATGGATTTGGTAATGATGGTGGTTATGGAGGAGGCGGCCCTGGTTACTCTGGAGGAAGCAGAGGCTATGGAAGTGGTGGACAGGGTTATGGAAACCAGGGCAGTGGCTATGGCGGGAGTGGCAGCTATGACAGCTATAACAACGGAGGCGGAGGCGGCTTTGGCGGTGGTAGTGGAAGCAATTTTGGAGGTGGTGGAAGCTACAATGATTTTGGGAATTACAACAATCAGTCTTCAAATTTTGGACCCATGAAGGGAGGAAATTTTGGAGGCAGAAGCTCTGGCCCCTATGGCGGTGGAGGCCAATACTTTGCAAAACCACGAAACCAAGGTGGCTATGGCGGTTCCAGCAGCAGCAGTAGCTATGGCAGTGGCAGAAGATTTTAA

hnRNPA1(del14-97aa)	ATGTCTAAGTCAGAGTCTCCTAAAGAGCCCGAACAGCTGCCAGGTGC<br>CCACTTAACTGTGAAAAAGATATTTGTTGGTGGCATTAAAGAAGACAC<br>TGAAGAACATCACCTAAGAGATTATTTTGAACAGTATGGAAAAATTGA<br>AGTGATTGAAATCATGACTGACCGAGGCAGTGGCAAGAAAAGGGGC<br>TTTGCCTTTGTAACCTTTGACGACCATGACTCCGTGGATAAGATTGTC<br>ATTCAGAAATACCATACTGTGAATGGCCACAACTGTGAAGTTAGAAA<br>AGCCCTGTCAAAGCAAGAGATGGCTAGTGCTTCATCCAGCCAAAGAG<br>GTCGAAGTGGTTCTGGAAACTTTGGTGGTGGTCGTGGAGGTGGTTTC<br>GGTGGGAATGACAACTTCGGTCGTGGAGGAAACTTCAGTGGTCGTGG<br>TGGCTTTGGTGGCAGCCGTGGTGGTGGTGGATATGGTGGCAGTGGGG<br>ATGGCTATAATGGATTTGGTAATGATGGTGGTTATGGAGGAGGCGGCC<br>CTGGTTACTCTGGAGGAAGCAGAGGCTATGGAAGTGGTGGACAGGGT<br>TATGGAAACCAGGGCAGTGGCTATGGCGGGAGTGGCAGCTATGACAG<br>CTATAACAACGGAGGCGGAGGCGGCTTTGGCGGTGGTAGTGGAAGCA<br>ATTTTGGAGGTGGTGGAAGCTACAATGATTTTGGGAATTACAACAATC<br>AGTCTTCAAATTTTGGACCCATGAAGGGAGGAAATTTTGGAGGCAGA<br>AGCTCTGGCCCCTATGGCGGTGGAGGCCAATACTTTGCAAAACCACG<br>AAACCAAGGTGGCTATGGCGGTTCCAGCAGCAGCAGTAGCTATGGCA<br>GTGGCAGAAGATTTTAA
hnRNPA1(del14-184aa)	ATGTCTAAGTCAGAGTCTCCTAAAGAGCCCGAACAGCTGGAGATGGC<br>TAGTGCTTCATCCAGCCAAAGAGGTCGAAGTGGTTCTGGAAACTTTG<br>GTGGTGGTCGTGGAGGTGGTTTCGGTGGGAATGACAACTTCGGTCGT<br>GGAGGAAACTTCAGTGGTCGTGGTGGCTTTGGTGGCAGCCGTGGTGG<br>TGGTGGATATGGTGGCAGTGGGGATGGCTATAATGGATTTGGTAATGA<br>TGGTGGTTATGGAGGAGGCGGCCCTGGTTACTCTGGAGGAAGCAGAG<br>GCTATGGAAGTGGTGGACAGGGTTATGGAAACCAGGGCAGTGGCTAT<br>GGCGGGAGTGGCAGCTATGACAGCTATAACAACGGAGGCGGAGGCG<br>GCTTTGGCGGTGGTAGTGGAAGCAATTTTGGAGGTGGTGGAAGCTAC<br>AATGATTTTGGGAATTACAACAATCAGTCTTCAAATTTTGGACCCATG<br>AAGGGAGGAAATTTTGGAGGCAGAAGCTCTGGCCCCTATGGCGGTGG<br>AGGCCAATACTTTGCAAAACCACGAAACCAAGGTGGCTATGGCGGTT<br>CCAGCAGCAGCAGTAGCTATGGCAGTGGCAGAAGATTTTAA

G3BP1(del11-113aa)	ATGGTGATGGAGAAGCCTAGTCCCCTGCTGCTTGCTCCTGAGGGGTCT GTTGCAAATAAATTCTATGTTCACAATGATATCTTCAGATACCAAGATG AGGTCTTTGGTGGGTTTGTCACTGAGCCTCAGGAGGAGTCTGAAGAA GAAGTAGAGGAACCTGAAGAAAGACAGCAAACACCTGAGGTGGTAC CTGATGATTCTGGAACTTTCTATGATCAGGCAGTTGTCAGTAATGACAT GGAAGAACATTTAGAGGAGCCTGTTGCTGAACCAGAGCCTGATCCTG AACCAGAACCAGAACAAGAACCTGTATCTGAAATCCAAGAGGAAAA GCCTGAGCCAGTATTAGAAGAAACTGCCCCTGAGGATGCTCAGAAGA GTTCTTCTCCAGCACCTGCAGACATAGCTCAGACAGTACAGGAAGAC TTGAGGACATTTTCTTGGGCATCTGTGACCAGTAAGAATCTTCCACCC AGTGGAGCTGTTCCAGTTACTGGGATACCACCTCATGTTGTTAAAGTA CCAGCTTCACAGCCCCGTCCAGAGTCTAAGCCTGAATCTCAGATTCCA CCACAAAGACCTCAGCGGGATCAAAGAGTGCGAGAACAACGAATAA ATATTCCTCCCCAAAGGGGACCCAGACCAATCCGTGAGGCTGGTGAG CAAGGTGACATTGAACCCCGAAGAATGGTGAGACACCCTGACAGTC ACCAACTCTTCATTGGCAACCTGCCTCATGAAGTGGACAAATCAGAG CTTAAAGATTTCTTTCAAAGTTATGGAAACGTGGTGGAGTTGCGCATT AACAGTGGTGGGAAATTACCCAATTTTGGTTTTGTTGTGTTTGATGAT TCTGAGCCTGTTCAGAAAGTCCTTAGCAACAGGCCCATCATGTTCAG AGGTGAGGTCCGTCTGAATGTCGAAGAGAAGAAGACTCGAGCTGCC AGGGAAGGCGACCGACGAGATAATCGCCTTCGGGGACCTGGAGGCC CTCGAGGTGGGCTGGGTGGTGGAATGAGAGGCCCTCCCCGTGGAGG CATGGTGCAGAAACCAGGATTTGGAGTGGGAAGGGGGCTTGCGCCA CGGCAGTGA
G3BP1(del142-225aa)	ATGGTGATGGAGAAGCCTAGTCCCCTGCTGGTCGGGCGGGAATTTGT GAGACAGTATTACACACTGCTGAACCAGGCCCCAGACATGCTGCATA GATTTTATGGAAAGAACTCTTCTTATGTCCATGGGGGATTGGATTCAA ATGGAAAGCCAGCAGATGCAGTCTACGGACAGAAAGAAATCCACAG GAAAGTGATGTCACAAAACTTCACCAACTGCCACACCAAGATTCGCC ATGTTGATGCTCATGCCACGCTAAATGATGGTGTGGTAGTCCAGGTGA TGGGGCTTCTCTCTAACAACAACCAGGCTTTGAGGAGATTCATGCAA ACGTTTGTCCTTGCTCCTGAGGGGTCTGTTGCAAATAAATTCTATGTTC ACAATGATATCTTCAGATACCAAGATGAGGTCTTTGGTGGGTTTGATG CTCAGAAGAGTTCTTCTCCAGCACCTGCAGACATAGCTCAGACAGTA CAGGAAGACTTGAGGACATTTTCTTGGGCATCTGTGACCAGTAAGAA TCTTCCACCCAGTGGAGCTGTTCCAGTTACTGGGATACCACCTCATGT TGTTAAAGTACCAGCTTCACAGCCCCGTCCAGAGTCTAAGCCTGAAT CTCAGATTCCACCACAAAGACCTCAGCGGGATCAAAGAGTGCGAGA ACAACGAATAAATATTCCTCCCCAAAGGGGACCCAGACCAATCCGTG AGGCTGGTGAGCAAGGTGACATTGAACCCCGAAGAATGGTGAGACA CCCTGACAGTCACCAACTCTTCATTGGCAACCTGCCTCATGAAGTGG ACAAATCAGAGCTTAAAGATTTCTTTCAAAGTTATGGAAACGTGGTG GAGTTGCGCATTAACAGTGGTGGGAAATTACCCAATTTTGGTTTTGTT GTGTTTGATGATTCTGAGCCTGTTCAGAAAGTCCTTAGCAACAGGCCC ATCATGTTCAGAGGTGAGGTCCGTCTGAATGTCGAAGAGAAGAAGAC TCGAGCTGCCAGGGAAGGCGACCGACGAGATAATCGCCTTCGGGGAC CTGGAGGCCCTCGAGGTGGGCTGGGTGGTGGAATGAGAGGCCCTCC CCGTGGAGGCATGGTGCAGAAACCAGGATTTGGAGTGGGAAGGGGG CTTGCGCCACGGCAGTGA

G3BP1(del226-243aa)	ATGGTGATGGAGAAGCCTAGTCCCCTGCTGGTCGGGCGGGAATTTGT<br>GAGACAGTATTACACACTGCTGAACCAGGCCCCAGACATGCTGCATA<br>GATTTTATGGAAAGAACTCTTCTTATGTCCATGGGGGATTGGATTCAA<br>ATGGAAAGCCAGCAGATGCAGTCTACGGACAGAAAGAAATCCACAG<br>GAAAGTGATGTCACAAAACTTCACCAACTGCCACACCAAGATTCGCC<br>ATGTTGATGCTCATGCCACGCTAAATGATGGTGTGGTAGTCCAGGTGA<br>TGGGGCTTCTCTCTAACAACAACCAGGCTTTGAGGAGATTCATGCAA<br>ACGTTTGTCCTTGCTCCTGAGGGGTCTGTTGCAAATAAATTCTATGTTC<br>ACAATGATATCTTCAGATACCAAGATGAGGTCTTTGGTGGGTTTGTCA<br>CTGAGCCTCAGGAGGAGTCTGAAGAAGAAGTAGAGGAACCTGAAGA<br>AAGACAGCAAACACCTGAGGTGGTACCTGATGATTCTGGAACTTTCT<br>ATGATCAGGCAGTTGTCAGTAATGACATGGAAGAACATTTAGAGGAG<br>CCTGTTGCTGAACCAGAGCCTGATCCTGAACCAGAACCAGAACAAG<br>AACCTGTATCTGAAATCCAAGAGGAAAAGCCTGAGCCAGTATTAGAA<br>GAAACTGCCCCTGAGGAAGACTTGAGGACATTTTCTTGGGCATCTGT<br>GACCAGTAAGAATCTTCCACCCAGTGGAGCTGTTCCAGTTACTGGGA<br>TACCACCTCATGTTGTTAAAGTACCAGCTTCACAGCCCCGTCCAGAGT<br>CTAAGCCTGAATCTCAGATTCCACCACAAAGACCTCAGCGGGATCAA<br>AGAGTGCGAGAACAACGAATAAATATTCCTCCCCAAAGGGGACCCAG<br>ACCAATCCGTGAGGCTGGTGAGCAAGGTGACATTGAACCCCGAAGA<br>ATGGTGAGACACCCTGACAGTCACCAACTCTTCATTGGCAACCTGCC<br>TCATGAAGTGGACAAATCAGAGCTTAAAGATTTCTTTCAAAGTTATGG<br>AAACGTGGTGGAGTTGCGCATTAACAGTGGTGGGAAATTACCCAATT<br>TTGGTTTTGTTGTGTTTGATGATTCTGAGCCTGTTCAGAAAGTCCTTA<br>GCAACAGGCCCATCATGTTCAGAGGTGAGGTCCGTCTGAATGTCGAA<br>GAGAAGAAGACTCGAGCTGCCAGGGAAGGCGACCGACGAGATAATC<br>GCCTTCGGGGACCTGGAGGCCCTCGAGGTGGGCTGGGTGGTGGAAT<br>GAGAGGCCCTCCCCGTGGAGGCATGGTGCAGAAACCAGGATTTGGA<br>GTGGGAAGGGGGCTTGCGCCACGGCAGTGA

G3BP1(del340-415aa)	ATGGTGATGGAGAAGCCTAGTCCCCTGCTGGTCGGGCGGGAATTTGT<br>GAGACAGTATTACACACTGCTGAACCAGGCCCCAGACATGCTGCATA<br>GATTTTATGGAAAGAACTCTTCTTATGTCCATGGGGGATTGGATTCAA<br>ATGGAAAGCCAGCAGATGCAGTCTACGGACAGAAAGAAATCCACAG<br>GAAAGTGATGTCACAAAACTTCACCAACTGCCACACCAAGATTCGCC<br>ATGTTGATGCTCATGCCACGCTAAATGATGGTGTGGTAGTCCAGGTGA<br>TGGGGCTTCTCTCTAACAACAACCAGGCTTTGAGGAGATTCATGCAA<br>ACGTTTGTCCTTGCTCCTGAGGGGTCTGTTGCAAATAAATTCTATGTTC<br>ACAATGATATCTTCAGATACCAAGATGAGGTCTTTGGTGGGTTTGTCA<br>CTGAGCCTCAGGAGGAGTCTGAAGAAGAAGTAGAGGAACCTGAAGA<br>AAGACAGCAAACACCTGAGGTGGTACCTGATGATTCTGGAACTTTCT<br>ATGATCAGGCAGTTGTCAGTAATGACATGGAAGAACATTTAGAGGAG<br>CCTGTTGCTGAACCAGAGCCTGATCCTGAACCAGAACCAGAACAAG<br>AACCTGTATCTGAAATCCAAGAGGAAAAGCCTGAGCCAGTATTAGAA<br>GAAACTGCCCCTGAGGATGCTCAGAAGAGTTCTTCTCCAGCACCTGC<br>AGACATAGCTCAGACAGTACAGGAAGACTTGAGGACATTTTCTTGGG<br>CATCTGTGACCAGTAAGAATCTTCCACCCAGTGGAGCTGTTCCAGTTA<br>CTGGGATACCACCTCATGTTGTTAAAGTACCAGCTTCACAGCCCCGTC<br>CAGAGTCTAAGCCTGAATCTCAGATTCCACCACAAAGACCTCAGCGG<br>GATCAAAGAGTGCGAGAACAACGAATAAATATTCCTCCCCAAAGGGG<br>ACCCAGACCAATCCGTGAGGCTGGTGAGCAAGGTGACATTGAACCCC<br>GAAGAATGGTGAGACACCCTGACAGTCGAGCTGCCAGGGAAGGCGA<br>CCGACGAGATAATCGCCTTCGGGGACCTGGAGGCCCTCGAGGTGGGC<br>TGGGTGGTGGAATGAGAGGCCCTCCCCGTGGAGGCATGGTGCAGAA<br>ACCAGGATTTGGAGTGGGAAGGGGGCTTGCGCCACGGCAGTGA

G3BP1(del410-466aa)	ATGGTGATGGAGAAGCCTAGTCCCCTGCTGGTCGGGCGGGAATTTGT<br>GAGACAGTATTACACACTGCTGAACCAGGCCCCAGACATGCTGCATA<br>GATTTTATGGAAAGAACTCTTCTTATGTCCATGGGGGATTGGATTCAA<br>ATGGAAAGCCAGCAGATGCAGTCTACGGACAGAAAGAAATCCACAG<br>GAAAGTGATGTCACAAAACTTCACCAACTGCCACACCAAGATTCGCC<br>ATGTTGATGCTCATGCCACGCTAAATGATGGTGTGGTAGTCCAGGTGA<br>TGGGGCTTCTCTCTAACAACAACCAGGCTTTGAGGAGATTCATGCAA<br>ACGTTTGTCCTTGCTCCTGAGGGGTCTGTTGCAAATAAATTCTATGTTC<br>ACAATGATATCTTCAGATACCAAGATGAGGTCTTTGGTGGGTTTGTCA<br>CTGAGCCTCAGGAGGAGTCTGAAGAAGAAGTAGAGGAACCTGAAGA<br>AAGACAGCAAACACCTGAGGTGGTACCTGATGATTCTGGAACTTTCT<br>ATGATCAGGCAGTTGTCAGTAATGACATGGAAGAACATTTAGAGGAG<br>CCTGTTGCTGAACCAGAGCCTGATCCTGAACCAGAACCAGAACAAG<br>AACCTGTATCTGAAATCCAAGAGGAAAAGCCTGAGCCAGTATTAGAA<br>GAAACTGCCCCTGAGGATGCTCAGAAGAGTTCTTCTCCAGCACCTGC<br>AGACATAGCTCAGACAGTACAGGAAGACTTGAGGACATTTTCTTGGG<br>CATCTGTGACCAGTAAGAATCTTCCACCCAGTGGAGCTGTTCCAGTTA<br>CTGGGATACCACCTCATGTTGTTAAAGTACCAGCTTCACAGCCCCGTC<br>CAGAGTCTAAGCCTGAATCTCAGATTCCACCACAAAGACCTCAGCGG<br>GATCAAAGAGTGCGAGAACAACGAATAAATATTCCTCCCCAAAGGGG<br>ACCCAGACCAATCCGTGAGGCTGGTGAGCAAGGTGACATTGAACCCC<br>GAAGAATGGTGAGACACCCTGACAGTCACCAACTCTTCATTGGCAAC<br>CTGCCTCATGAAGTGGACAAATCAGAGCTTAAAGATTTCTTTCAAAGT<br>TATGGAAACGTGGTGGAGTTGCGCATTAACAGTGGTGGGAAATTACC<br>CAATTTTGGTTTTGTTGTGTTTGATGATTCTGAGCCTGTTCAGAAAGT<br>CCTTAGCAACAGGCCCATCATGTTCAGAGGTGAGGTCCGTCTGAATTG<br>A
G3BP1(del340-466aa)	ATGGTGATGGAGAAGCCTAGTCCCCTGCTGGTCGGGCGGGAATTTGT<br>GAGACAGTATTACACACTGCTGAACCAGGCCCCAGACATGCTGCATA<br>GATTTTATGGAAAGAACTCTTCTTATGTCCATGGGGGATTGGATTCAA<br>ATGGAAAGCCAGCAGATGCAGTCTACGGACAGAAAGAAATCCACAG<br>GAAAGTGATGTCACAAAACTTCACCAACTGCCACACCAAGATTCGCC<br>ATGTTGATGCTCATGCCACGCTAAATGATGGTGTGGTAGTCCAGGTGA<br>TGGGGCTTCTCTCTAACAACAACCAGGCTTTGAGGAGATTCATGCAA<br>ACGTTTGTCCTTGCTCCTGAGGGGTCTGTTGCAAATAAATTCTATGTTC<br>ACAATGATATCTTCAGATACCAAGATGAGGTCTTTGGTGGGTTTGTCA<br>CTGAGCCTCAGGAGGAGTCTGAAGAAGAAGTAGAGGAACCTGAAGA<br>AAGACAGCAAACACCTGAGGTGGTACCTGATGATTCTGGAACTTTCT<br>ATGATCAGGCAGTTGTCAGTAATGACATGGAAGAACATTTAGAGGAG<br>CCTGTTGCTGAACCAGAGCCTGATCCTGAACCAGAACCAGAACAAG<br>AACCTGTATCTGAAATCCAAGAGGAAAAGCCTGAGCCAGTATTAGAA<br>GAAACTGCCCCTGAGGATGCTCAGAAGAGTTCTTCTCCAGCACCTGC<br>AGACATAGCTCAGACAGTACAGGAAGACTTGAGGACATTTTCTTGGG<br>CATCTGTGACCAGTAAGAATCTTCCACCCAGTGGAGCTGTTCCAGTTA<br>CTGGGATACCACCTCATGTTGTTAAAGTACCAGCTTCACAGCCCCGTC<br>CAGAGTCTAAGCCTGAATCTCAGATTCCACCACAAAGACCTCAGCGG<br>GATCAAAGAGTGCGAGAACAACGAATAAATATTCCTCCCCAAAGGGG<br>ACCCAGACCAATCCGTGAGGCTGGTGAGCAAGGTGACATTGAACCCC<br>GAAGAATGGTGAGACACCCTGACAGTTGA

h-Ubiquitin	ATGTACCCATACGATGTTCCAGATTACGCTATGCAGATCTTCGTGAAGACCCTGACTGGTAAGACCATCACTCTCGAAGTGGAGCCGAGTGACACCATTGAGAATGTCAAGGCAAAGATCCAAGACAAGGAAGGCATCCCTCCTGACCAGCAGAGGTTGATCTTTGCTGGGAAACAGCTGGAAGATGGACGCACCCTGTCTGACTACAACATCCAGAAAGAGTCCACCCTGCACCTGGTCCTCCGTCTCAGAGGTGGGTAG
